# Topical *Dunaliella salina*-Derived Exosome Loaded with Methotrexate Alleviates Psoriasis-like Inflammation via STAT3-Dependent Th17/Treg Balance

**DOI:** 10.3390/pharmaceutics18091085

**Published:** 2026-08-28

**Authors:** Yitong Yang, Dandan Guo, Wei Chen, Binbin Sun, Mengyu Qiu, Kai Wang, Wenbo Dou, Kang Wang, Zhanjiang Zhang, Shuying Feng

**Affiliations:** 1 Medical College, Henan University of Chinese Medicine, Zhengzhou 450046, China; 2024815089@hactcm.edu.cn (Y.Y.); gdd461900959@hactcm.edu.cn (D.G.); chenwei@hactcm.edu.cn (W.C.); 2024702008@hactcm.edu.cn (B.S.); 2024702012@hactcm.edu.cn (M.Q.); 2024702013@hactcm.edu.cn (K.W.); 2025702001@hactcm.edu.cn (W.D.); 2022150050@hactcm.edu.cn (K.W.); 2 Henan Engineering Research Center for Chinese Medicine Foods for Special Medical Purpose, Zhengzhou 450046, China; 3 Medical Plant Garden of Guangxi Zhuang Autonomous Region, Nanning 530023, China

**Keywords:** *Dunaliella salina* exosome, psoriasis, topical administration, STAT3 signal pathway, Th17/Treg immune balance

## Abstract

**Background**: Methotrexate (MTX) is a well-established therapeutic agent for psoriasis owing to its anti-inflammatory and immunomodulatory effects. However, its clinical use is limited by insufficient local accumulation in skin lesions and the potential risk of systemic exposure. *Dunaliella salina*-derived exosome (DsEXO) has favorable biocompatibility, low immunogenicity and potential skin delivery capacity, making it a promising natural nanocarrier for topical MTX delivery. This study aimed to construct *Dunaliella salina*-derived exosome loaded with methotrexate (DsEXO@MTX) and evaluate its therapeutic efficacy and potential mechanisms in psoriasis-like skin inflammation. **Methods**: DsEXO@MTX was prepared and characterized in terms of morphology, particle size, surface charge and drug-loading capacity. Cellular uptake, skin retention and tissue distribution were evaluated using fluorescence imaging and skin section analysis. Therapeutic efficacy was evaluated in an imiquimod-induced psoriasis-like mouse model by clinical scoring, histopathological examination, spleen index measurement and Ki-67 immunofluorescence staining. STAT3 phosphorylation and Th17/Treg differentiation were further examined to explore the potential immunomodulatory mechanism. **Results**: DsEXO@MTX exhibited a relatively uniform particle size distribution and drug-loading capacity. In vivo fluorescence imaging and skin section analysis showed that DsEXO@MTX enhanced local skin retention and promoted fluorescence distribution in epidermal and dermal regions. It significantly alleviated IMQ-induced erythema, scaling, epidermal thickening, inflammatory infiltration, splenomegaly and abnormal keratinocyte proliferation in psoriasis-like mice. Mechanistically, DsEXO@MTX reduced STAT3 phosphorylation and modulated Th17/Treg differentiation, suggesting restoration of immune balance in psoriatic inflammation. **Conclusions**: DsEXO@MTX represents a natural exosome-like nanovesicle-mediated topical MTX delivery system that improves local drug delivery and enhances therapeutic efficacy in IMQ-induced psoriasis-like skin inflammation, particularly when administered topically. These findings provide a potential strategy for safer and more efficient local treatment of psoriasis.

## 1. Introduction

Psoriasis is a chronic, recurrent, immune-mediated inflammatory skin disorder characterized by erythematous plaques, scaling, epidermal hyperplasia and pruritus [1,2]. In addition to cutaneous manifestations, psoriasis is frequently associated with systemic comorbidities, including psoriatic arthritis, obesity, metabolic syndrome and cardiovascular diseases, which markedly reduce patients’ quality of life [3,4]. A growing body of evidence supports that psoriasis extends beyond a localized cutaneous inflammation to a systemic immune-mediated disorder, driven by the complex interplay of genetic susceptibility, environmental triggers, immune dysregulation and keratinocyte dysfunction [3,5]. During disease progression, dendritic cells, macrophages, T cells and keratinocytes orchestrate a persistent, self-amplifying inflammatory network [5,6]. Among these interactions, the TNF-α/IL-23/IL-17 axis represents a pivotal signaling cascade that contributes to disease initiation, inflammatory amplification and the maintenance of psoriatic lesions [7]. Inflammatory mediators, including IL-17, IL-22 and TNF-α, further induce keratinocyte hyperproliferation, abnormal differentiation and chemokine production, thereby promoting continuous immune-cell recruitment into lesional skin [8]. Therefore, developing therapeutic strategies that effectively inhibit local inflammation and keratinocyte hyperproliferation while reducing long-term treatment-related adverse effects remains a major focus in psoriasis management.

The STAT3 signaling pathway is critically involved in inflammatory amplification, keratinocyte activation and T-cell differentiation during disease progression [9,10]. Inflammatory cytokines, including IL-6 and IL-23, can induce STAT3 phosphorylation and subsequently drive Th17 cell differentiation, leading to increased production of inflammatory effector molecules such as IL-17A and IL-17F [11]. In parallel, aberrant STAT3 activation impairs Treg cell stability or function, thereby shifting the Th17/Treg immune balance toward a pro-inflammatory phenotype [11,12]. The imbalance between Th17 and Treg cells is a key immunological hallmark of psoriasis and is closely linked to keratinocyte hyperproliferation, cytokine overproduction and the persistence of psoriatic lesions [11,12]. These findings suggest that STAT3-mediated Th17/Treg imbalance may represent an important mechanistic link between immune activation and persistent psoriatic inflammation.

Methotrexate (MTX) remains one of the most commonly used conventional systemic agents for treatment of moderate-to-severe psoriasis, owing to its well-documented anti-inflammatory, anti-proliferative and immunomodulatory activities [13,14]. It exerts therapeutic effects by interfering with folate metabolism, suppressing purine and pyrimidine synthesis, promoting adenosine-mediated anti-inflammatory signaling and reducing the production of multiple pro-inflammatory cytokines, thereby alleviating psoriatic skin lesions and systemic inflammation [14]. Nevertheless, the clinical utility of MTX is substantially compromised by systemic adverse effects inherent to conventional administration routes. Topical treatment remains an important component of psoriasis management, particularly for localized diseases [15]. Although topical delivery represents a more rational alternative by concentrating drug exposure at lesional sites while minimizing systemic toxicity [16,17], this approach remains challenged by impaired penetration across the psoriatic skin barrier, insufficient retention within lesional tissues, inadequate delivery to viable epidermal and dermal target cells and formulation-related limitations of topical immunosuppressive agents [18,19]. Accordingly, there is an urgent need to develop an advanced delivery system capable of overcoming these barriers to enhance local MTX accumulation in psoriatic lesions, improve therapeutic utilization and mitigate systemic adverse effects [20].

Extracellular vesicles (EVs) are lipid-bilayer-delimited particles released by cells and involved in intercellular communication [21]. Their ability to transport biological and therapeutic cargoes has attracted considerable interest in their development as drug-delivery platforms [22]. However, the clinical translation of conventional mammalian cell-derived EVs remains limited by restricted source availability, low production yield, biological heterogeneity and challenges in scalable manufacturing and quality control [23]. In contrast, microalgae-derived extracellular vesicles and exosome-like nanovesicles offer advantages associated with renewable source materials and scalable cultivation and have attracted increasing attention as naturally derived therapeutic carriers [23,24]. EVs have also been investigated as mediators and potential therapeutic tools in psoriasis [25].

Recent studies have expanded the application of *Dunaliella salina*-derived exosome-like nanovesicles in therapeutic delivery. Wei et al. developed engineered *Dunaliella salina* exosome-like nanovesicle for the sequential delivery of miR-375 and anti-PD-L1 [26]. More recently, *Dunaliella salina*-derived small extracellular vesicles were used to load oridonin for colorectal cancer therapy [27]. MTX loading has also been investigated using mammalian cell-derived EVs, supporting the feasibility of EV-mediated MTX delivery [28]. However, MTX loading into *Dunaliella salina*-derived vesicles and its application in topical psoriasis treatment have not been systematically investigated.

In the present study, *Dunaliella salina*-derived exosome loaded with methotrexate (DsEXO@MTX) was constructed as a natural nanovesicle-mediated topical delivery system for psoriasis therapy. The physicochemical properties of DsEXO@MTX, including morphology, particle size, zeta potential and drug-loading performance, were first systematically characterized. Its skin delivery capacity was then evaluated by in vivo fluorescence imaging, frozen skin section analysis and cellular uptake assays. Using an IMQ-induced psoriasis-like dermatitis mouse model, we further compared the therapeutic effects of topical and oral administration of DsEXO@MTX on lesion severity, epidermal hyperplasia, keratinocyte proliferation and spleen-associated immune alterations. Additionally, potential mechanisms were investigated through RNA sequencing, flow-cytometric profiling of cutaneous CD4^+^ T cell subsets, in vitro Th17/Treg differentiation assays and assessment of STAT3 phosphorylation. These findings provide experimental evidence supporting the optimization of local MTX delivery, the expansion of DsEXO functional applications and the development of safe and efficient nanotherapeutic strategies for psoriasis treatment.

## 2. Materials and Methods

### 2.1. Materials

#### 2.1.1. Cell Lines and Reagents

The human spontaneously immortalized keratinocyte cell line HaCaT was obtained from Procell Life Science & Technology Co., Ltd. (Wuhan, China; Cat. No. CL-0090; Cellosaurus accession No. CVCL_0038; RRID: CVCL_0038). Dulbecco’s modified Eagle’s medium (DMEM; #C11995500BT; Gibco, Grand Island, NY, USA), fetal bovine serum (FBS; #10099141C; Gibco, Grand Island, NY, USA) and penicillin-streptomycin solution (#15140122; Gibco, Grand Island, NY, USA) were used for cell culture. DAPI solution (1 mg/mL; #62248), Pierce™ 16% Formaldehyde (*w*/*v*), methanol-free (#28908) and ProLong™ Gold Antifade Mountant (#P36930) were purchased from Thermo Fisher Scientific (Waltham, MA, USA). PKH26 Red Fluorescent Cell Linker Kit (#PKH26GL; Sigma-Aldrich, St. Louis, MO, USA) was used for fluorescence labeling and imaging. Pierce™ BCA Protein Assay Kit (#23227), 0.22 μm syringe filters (#SLGP033RB; Millipore, Burlington, MA, USA) and phosphate-buffered saline (PBS; #10010023; Gibco, Grand Island, NY, USA) were used for DsEXO isolation and characterization. Imiquimod (IMQ) cream (National Medical Products Administration approval No. H20030128; Mingxin Pharmaceutical Co., Ltd., Chengdu, China) and methotrexate (MTX; #M8407; Sigma-Aldrich, St. Louis, MO, USA) were used in this study.

#### 2.1.2. Reagents for Skin Digestion and Flow Cytometry

Collagenase IV (#C5138; Sigma-Aldrich, St. Louis, MO, USA), Dispase II (neutral protease, grade II; #04942078001; Roche Diagnostics GmbH, Mannheim, Germany), DNase I (#DN25; Sigma-Aldrich, St. Louis, MO, USA) and 70 μm cell strainers (#352350; Corning Incorporated, Corning, NY, USA) were used to prepare single-cell suspensions from mouse dorsal skin tissues. For flow cytometry, the following antibodies and reagents were used: anti-CD3 (#553061), anti-CD4 (#563151), anti-IL-17A (#559502), anti-Foxp3 (#563101), anti-IFN-γ (#561040), anti-IL-4 (#554436), anti-mouse CD16/CD32 Fc Block (#553142), BD Pharmingen™ Transcription Factor Buffer Set (#562574), BD Pharm Lyse™ Lysing Buffer (10×) (#555899), BD Pharmingen™ Leukocyte Activation Cocktail with BD GolgiPlug™ (#550583), BD Horizon™ Fixable Viability Stain 780 (#565388) and BD Pharmingen™ Stain Buffer (FBS) (#554656) were purchased from BD Pharmingen (San Diego, CA, USA). The anti-CD45 (#103138; BioLegend, San Diego, CA, USA), FITC-conjugated anti-phospho-STAT3 (Tyr705) antibody (#11-9033-42; Invitrogen, Thermo Fisher Scientific, Waltham, MA, USA) and anti-CD25 (#561038; BD Pharmingen, San Diego, CA, USA) were purchased from different companies.

### 2.2. Construction of DsEXO@MTX

#### 2.2.1. Culture of Ds Cells

*Dunaliella salina* cells were cultured and clonally expanded according to a previously established protocol from our group, with minor modifications [26,27]. *Dunaliella salina* (Ds) strain CCAP19/18 was obtained from Guangyu Biotechnology Co., Ltd. (Wenzhou, China). Cryopreserved Ds cells were rapidly thawed in a 37 °C water bath and then the cell suspension was carefully transferred into PKS liquid medium and cultured overnight at 26 °C under a 14:10 h light/dark cycle. On the following day, the cell density was estimated using a NanoDrop 2000 spectrophotometer (Thermo Fisher Scientific, Wilmington, DE, USA). Subsequently, 50 μL of cell suspension at a density of 1 × 10^6^ cells/mL was evenly inoculated onto the surface of PKS solid medium and cultured under the same conditions for 7–10 d. Under aseptic conditions in a biosafety cabinet, a well-grown single algal colony was selected from the solid medium and transferred onto fresh PKS solid medium for further culture to obtain purified clonal algal cultures. A purified single algal colony was then picked and inoculated into PKS liquid medium for several days until the cell density reached 1 × 10^6^ cells/mL. Finally, Ds cultures in the logarithmic growth phase were collected for subsequent DsEXO isolation.

#### 2.2.2. Isolation of DsEXO

In this study, the term DsEXO refers to exosome-like nanovesicles derived from the culture supernatant of *Dunaliella salina*, rather than classical exosomes strictly defined in mammalian cells. DsEXO were isolated from the culture supernatant using differential centrifugation combined with ultracentrifugation, adapted from previously published protocols with minor modifications [26,27]. Briefly, Ds culture medium was maintained at 4 °C during processing and first centrifuged at 2000 *g* for 10 min to remove intact algal cells. The resulting supernatant was collected and centrifuged at 10,000 *g* for 30 min to further eliminate cell debris, large extracellular particles and vesicles. After centrifugation, the supernatant was collected and filtered through a 0.22 μm filter membrane to remove residual large particles. The filtrate was then subjected to ultracentrifugation at 118,000 *g* for 90 min at 4 °C to pellet exosome-like nanovesicles. After discarding the supernatant, the pellet was gently resuspended in pre-cooled sterile PBS and ultracentrifuged again at 118,000 *g* for 90 min at 4 °C to further remove soluble proteins and residual components. Finally, the supernatant was discarded and the pellet was collected and resuspended in an appropriate volume of pre-cooled sterile PBS to obtain the DsEXO suspension. The total protein concentration of DsEXO was determined using the BCA protein assay and the samples were aliquoted according to the requirements of subsequent experiments. To minimize the effects of repeated freeze–thaw cycles on vesicle structure and particle size distribution, all samples were aliquoted for single use and stored at −80 °C [29]. Repeated freeze–thaw cycles were avoided during storage. Before use in subsequent experiments, DsEXO samples were characterized in terms of particle size, zeta potential, and morphology to confirm their physicochemical characteristics. Samples were stored at −80 °C and used within 6 months of preparation.

#### 2.2.3. Preparation of DsEXO@MTX

DsEXO@MTX was prepared by electroporation. Owing to its limited and pH-dependent aqueous solubility, MTX was first dissolved in DMSO to prepare a 20 mg/mL stock solution. Subsequently, 50 μL of the MTX stock solution, corresponding to 1.0 mg of MTX, was mixed with 1.0 mg of DsEXO and diluted to a final volume of 1 mL with electroporation buffer containing 0.25 M sucrose, 1 mM MgCl_2_, and 10 mM HEPES (pH 7.4). The resulting loading mixture contained 1.0 mg/mL MTX, with an MTX-to-DsEXO feeding mass ratio of 1:1 and a final DMSO concentration of 5% (*v*/*v*). No visible precipitation was observed after dilution with the electroporation buffer.

The mixture was transferred to a 0.2 cm electroporation cuvette and subjected to a single electric pulse at 300 V and 100 μF. Following electroporation, the sample was incubated at 37 °C for 30 min to facilitate vesicle membrane resealing. Unencapsulated MTX was subsequently removed by ultracentrifugation at 118,000 *g* for 90 min at 4 °C. The resulting pellet was washed twice with PBS under the same centrifugation conditions and resuspended in an appropriate volume of PBS to obtain DsEXO@MTX. The formulation was aliquoted according to the requirements of subsequent experiments and stored at −80 °C until use. Repeated freeze–thaw cycles were avoided.

### 2.3. Identification and Characterization of DsEXO and DsEXO@MTX

#### 2.3.1. Observation of DsEXO and DsEXO@MTX by TEM

The morphology of DsEXO and DsEXO@MTX was observed using transmission electron microscopy (TEM). Briefly, 20 μL of DsEXO or DsEXO@MTX suspension was dropped onto a piece of parafilm and a copper grid was gently placed face-down onto the sample droplet for 5 min to allow sample adsorption. After adsorption, excess liquid was carefully removed using filter paper. Subsequently, 10 μL of 3% uranyl acetate staining solution was dropped onto a new piece of parafilm and the copper grid containing the adsorbed sample was placed onto the staining droplet for negative staining. After staining, the copper grid was air-dried at room temperature and then observed using TEM.

#### 2.3.2. Particle Size and Zeta Potential Analysis of DsEXO and DsEXO@MTX

The hydrodynamic diameter, polydispersity index (PDI), and zeta potential of DsEXO and DsEXO@MTX were measured using a BeNano 180 Zeta Pro nanoparticle size and zeta potential analyzer (Bettersize Instruments Ltd., Dandong, China) equipped with a 50 mW, 671 nm solid-state laser. Hydrodynamic diameter and PDI were determined by dynamic light scattering at a backscattering angle of 173° and a measurement temperature of 25 °C, whereas zeta potential was determined by electrophoretic light scattering using phase analysis light scattering technology. Before measurement, DsEXO and DsEXO@MTX suspensions were appropriately diluted with sterile 1× phosphate-buffered saline containing 155.17 mM NaCl, 2.97 mM Na_2_HPO_4_·7H_2_O, and 1.06 mM KH_2_PO_4_ (Gibco, Grand Island, NY, USA; Cat. No. 10010023; pH 7.4; without calcium, magnesium, or phenol red) and equilibrated at 25 °C. The diluted samples were transferred into the corresponding particle-size or zeta-potential cuvettes according to the manufacturer’s instructions. Each sample was measured in triplicate, and the results are presented as the mean ± standard deviation.

#### 2.3.3. FTIR Characterization

Fourier-transform infrared spectroscopy (FTIR) was used to characterize the functional groups of DsEXO, MTX, the physical mixture of DsEXO and MTX (DsEXO + MTX) and DsEXO@MTX. DsEXO and DsEXO@MTX samples were lyophilized to obtain dry powders, while MTX powder was used directly for FTIR analysis. For the physical mixture, lyophilized DsEXO and MTX powder were mixed according to the same feeding ratio used for DsEXO@MTX preparation before FTIR analysis. Each sample was thoroughly ground with dry potassium bromide (KBr) powder to obtain a homogeneous mixture, which was then compressed into transparent pellets. FTIR spectra were recorded over the wavenumber range of 4000–400 cm^−1^. The characteristic absorption bands of each sample were compared to evaluate functional group changes and potential interactions associated with MTX loading in DsEXO@MTX.

#### 2.3.4. Determination of Encapsulation Efficiency and Drug Loading Content

The encapsulation efficiency (EE) and drug loading content (DLC) of DsEXO@MTX were determined using an indirect HPLC method. DsEXO and MTX were mixed at an initial mass ratio of 1:1 and subjected to electroporation as described above. Following membrane recovery, the samples were ultracentrifuged to separate DsEXO@MTX from unencapsulated MTX. The resulting pellet was resuspended in pre-cooled PBS and subjected to two additional ultracentrifugation under the same conditions. The supernatants collected after the three ultracentrifugation steps were pooled, and the total volume was accurately measured. The concentration of unencapsulated MTX in the pooled supernatant was quantified by HPLC. The amount of free MTX was calculated by multiplying its concentration by the total supernatant volume, whereas the amount of loaded MTX was calculated by subtracting the free MTX amount from the initially added MTX amount. EE and DLC were calculated using the following equations:

$$EE(\%)=\frac{M_{MTX,initial}-M_{MTX,free}}{M_{MTX,initial}}\times 100$$

$$DLC(\%)=\frac{M_{MTX,loaded}}{M_{MTX,loaded}+M_{DsEXO,initial}}\times 100$$

where *M*_MTX,initial_ represents the initially added amount of MTX, *M*_MTX,free_ represents the amount of unencapsulated MTX detected in the pooled supernatant, *M*_MTX,loaded_ represents the indirectly calculated amount of MTX loaded into DsEXO, and *M*_DsEXO,initial_ represents the initial amount of DsEXO used for drug loading.

#### 2.3.5. Physicochemical Characterization of DsEXO After Storage at −80 °C

Aliquoted DsEXO samples stored at −80 °C for 3 months were subjected to physicochemical characterization. To minimize alterations associated with repeated freeze–thaw cycles, each aliquot was thawed only once at 4 °C and gently mixed by inversion without vortexing or sonication. The hydrodynamic diameter, polydispersity index (PDI), and zeta potential of the post-thaw DsEXO samples were measured under the same conditions described in Section 2.3.2. All samples were diluted with the same batch of PBS at an identical dilution factor and equilibrated at 25 °C before measurement.

### 2.4. Skin Delivery and Cellular Uptake Assays

#### 2.4.1. Skin Delivery Assay

FITC-labeled methotrexate (FITC-MTX) was used as a model fluorescent cargo to evaluate DsEXO-mediated skin delivery behavior of small-molecule drugs. PKH26 and FITC fluorescence labeling was used for qualitative tracking of vesicle-associated and MTX-related fluorescent signals, respectively [30]. One day before the experiment, the dorsal hair of C57BL/6J mice was removed to expose an administration area of approximately 2 cm × 2 cm. On the day of the experiment, mice were placed on a thermostatic plate and free FITC-MTX or FITC-MTX-loaded PKH26-labeled DsEXO (PKH26-DsEXO@FITC-MTX) was evenly applied to the depilated dorsal skin area. The dose, administration volume and application area used in the skin delivery assay were consistent with those used in the therapeutic experiment. FITC-MTX was used as a fluorescent substitute for MTX to enable visualization of cutaneous distribution. At 0, 2, 4 and 6 h after administration, the distribution and intensity of fluorescence signals at the administration site were continuously monitored in vivo using an imaging system to evaluate the local skin retention capacity of formulations. The excitation/emission wavelengths were set at 490/525 nm for FITC and 551/567 nm for PKH26. At 6 h after administration, mice were euthanized by CO_2_ inhalation and dorsal skin tissues from the administration site were excised. The skin samples were gently rinsed with cold PBS to remove residual surface formulation and fixed in 4% paraformaldehyde. After being embedded in OCT compound, 10 μm-thick serial frozen sections were prepared using a CryoStar NX50 cryostat microtome (Epredia Laboratory Products Manufacturing (Shanghai) Co., Ltd., Shanghai, China). The fluorescence distribution of FITC-MTX and PKH26-DsEXO in skin sections was observed using a Nikon Eclipse C1 upright fluorescence microscope (Nikon Corporation, Tokyo, Japan). Z-stack scanning was performed to visualize the spatial distribution of fluorescence signals within the skin tissues. Images were acquired using NIS-Elements software (version 5.20) and merged using AIpathwell software (version 2.0), and processed using CaseViewer software (version 2.4).

#### 2.4.2. Cellular Uptake Assay

HaCaT cells were seeded into confocal dishes at a density of 1 × 10^5^ cells/mL and cultured until the cell confluence reached approximately 50%. The original culture medium was then removed and cells were washed once with PBS. Subsequently, 1 mL of DMEM containing 10% fetal bovine serum was added. The cells were divided into two groups: the free FITC-MTX group and the PKH26-DsEXO@FITC-MTX group. The final concentration of FITC-MTX was maintained at 1 μg/mL in both groups. The cells were incubated at 37 °C in the dark for 6 h. After incubation, the treatment medium was discarded and the cells were washed three to five times with cold PBS for 3 min each time. After that, cells were fixed with 4% paraformaldehyde for 15 min and then stained with DAPI solution in the dark for 10 min. After washing three times with PBS, antifade mounting medium was added and the cells were observed using an FV1200 confocal laser scanning microscope (Olympus Corporation, Tokyo, Japan).

### 2.5. Animal Experiments

Female C57BL/6J mice aged 9 weeks and weighing 20 ± 2 g were purchased from SPF Biotechnology Co., Ltd. (Beijing, China; SCXK [Jing] 2024-0001) and housed in the Experimental Animal Center of Henan University of Chinese Medicine (Zhengzhou, China; approval No. SYXK [Yu] 2021-0015). This study was approved by the Animal Experimental Ethics Committee of Henan University of Chinese Medicine on 18 September 2025 (approval No. 1763; ethics No. 3255). After 1 week of acclimatization, mice were randomly divided into eight experimental groups, with five mice in each group: (1) control group (Con); (2) model group (Mod); (3) oral DsEXO group (DsEXO-O); (4) oral methotrexate group (MTX-O); (5) oral DsEXO@MTX group (DsEXO@MTX-O); (6) topical DsEXO group (DsEXO-T); (7) topical methotrexate group (MTX-T) and (8) topical DsEXO@MTX group (DsEXO@MTX-T).

One day prior to the experiment, dorsal hair was removed from isoflurane-anesthetized mice over an approximately 2 cm × 2 cm area, after which psoriasis-like dermatitis was induced by daily topical application of 62.5 mg IMQ cream (or Vaseline for controls) for 6 consecutive days [31], with corresponding treatments administered 6 h post-IMQ application. In all MTX-treated groups, MTX was dosed at 1 mg/kg. Based on the approximately 20% estimated drug loading content determined by HPLC, DsEXO@MTX was administered at 5 mg/kg, corresponding to 1 mg/kg MTX and 4 mg/kg DsEXO carrier. DsEXO-alone groups received an equivalent DsEXO dose of 4 mg/kg. Oral groups received 100 μL by gavage, while topical groups received 100 μL evenly applied to the 2 cm × 2 cm dorsal lesional area. After completion of final treatment on day 6, mice were euthanized by cervical dislocation under anesthesia and dorsal skin tissues were collected for histological and immunohistochemical analyses after fixation in 4% PFA or stored at −80 °C for molecular assays.

### 2.6. Psoriasis Area and Severity Index Scoring

The severity of dorsal skin lesions in mice was evaluated using a modified psoriasis area and severity index (PASI) scoring system before daily treatment [31]. The scoring items included erythema, scaling and skin thickening. Each item was scored from 0 to 4 as follows: 0 = none, 1 = mild, 2 = moderate, 3 = severe and 4 = very severe. The total score was calculated as the sum of three individual scores, ranging from 0 to 12. A higher total score indicated more severe psoriasis-like skin lesions.

### 2.7. Spleen Index

After completion of final treatment on day 6, mice were euthanized by cervical dislocation under anesthesia. Body weight was recorded and the spleen was carefully excised. The spleen was gently rinsed with PBS to remove residual blood from the surface, blotted dry with filter paper and weighed using an analytical balance. The spleen index was calculated according to the following formula:

Spleen index = spleen weight (mg)/body weight (g)

### 2.8. Histological Analysis

#### 2.8.1. Hematoxylin and Eosin Staining

After completion of final treatment on day 6, mice were euthanized under anesthesia and dorsal skin tissues from the treated area, approximately 1.0 cm × 0.5 cm in size, were collected and immediately fixed in 4% paraformaldehyde for 48 h. The fixed tissues were dehydrated through a graded ethanol series, including 70%, 80%, 95% and 100% ethanol for 1 h each, followed by clearing in xylene and embedding in paraffin. Serial sections with a thickness of 4–5 μm were then prepared using a microtome. After routine deparaffinization and rehydration, the sections were stained with hematoxylin for 5–8 min, rinsed with running water and blued. Subsequently, the sections were stained with eosin for 2–3 min. After staining, the sections were dehydrated through a graded ethanol series, cleared in xylene and mounted with neutral balsam. Histopathological changes in the skin tissues were observed under a light microscope.

#### 2.8.2. Ki-67 Immunofluorescence Staining

Paraffin-embedded skin tissue sections with a thickness of 4–5 μm were routinely deparaffinized and rehydrated. Antigen retrieval was performed using 0.01 M sodium citrate buffer (pH 6.0) by microwave heating. Briefly, the sections were heated at high power until boiling, maintained for 10 min and then naturally cooled to room temperature. After washing with PBS, the sections were blocked with 5% bovine serum albumin (BSA) for 30 min. After removal of blocking solution, sections were incubated with rabbit anti-Ki-67 monoclonal antibody [SP6] (#ab16667; Abcam, Cambridge, UK; 1:200) in a humidified chamber at 4 °C overnight. After washing with PBS, the sections were incubated with Alexa Fluor™ 488-conjugated goat anti-rabbit IgG (H + L) secondary antibody (#A-11008; Invitrogen, Thermo Fisher Scientific, Waltham, MA, USA; 1:200) in the dark for 1 h. After further washing with PBS, the sections were mounted with ProLong™ Gold Antifade Mountant with DAPI. For negative control, PBS was used in place of the primary antibody. Images were acquired using Nikon Eclipse C1 upright fluorescence microscope. Five non-overlapping fields were randomly selected from each section at ×200 magnification. The percentage of Ki-67-positive cells among total epidermal cells was calculated using ImageJ software (version 1.54) and defined as the proliferation index.

### 2.9. RNA Sequencing and Bioinformatic Analysis

Total RNA was extracted from dorsal skin tissues using TRIzol reagent (Invitrogen, Carlsbad, CA, USA) according to the manufacturer’s instructions. Skin samples from the Con, Mod, DsEXO-T, MTX-T and DsEXO@MTX-T groups were used for RNA sequencing, with three biological replicates included in each group. Only high-quality RNA samples with an OD260/280 ratio of 1.8–2.1 and RNA integrity number (RIN) ≥ 7.0 were used for library construction. First-strand cDNA was synthesized using random hexamer primers, followed by second-strand cDNA synthesis. The double-stranded cDNA was purified using AMPure XP beads and subjected to end repair, A-tailing, adapter ligation, fragment size selection and PCR amplification. The quality of constructed libraries was assessed using a Qubit 2.0 Fluorometer (Invitrogen, Carlsbad, CA, USA) and an Agilent 2100 Bioanalyzer (Agilent Technologies, Santa Clara, CA, USA) and the effective library concentration was determined by quantitative PCR. Qualified libraries were sequenced on the DNBSEQ-T7RS platform using a paired-end 150 bp sequencing strategy. The resulting clean reads were aligned to the mouse reference genome GRCm39 using HISAT2 v2.1.0 [32]. Gene expression levels were quantified as fragments per kilobase of transcript per million mapped reads (FPKM) using StringTie v2.1.3b [33] for expression visualization and descriptive analysis. Differentially expressed genes (DEGs) were identified using DESeq2 v1.26.0 [34] based on raw read count matrices, with thresholds of |log_2_ fold change| ≥ 1 and false discovery rate (FDR) < 0.05. To evaluate the disease-reversal effect of different treatments, disease-associated DEGs were first identified by comparing the Mod group with the Con group. Genes upregulated in the Mod group but significantly downregulated after treatment, as well as genes downregulated in the Mod group but significantly upregulated after treatment, were defined as reversed genes. The reversal ratio was calculated as the number of reversed disease-associated genes divided by the total number of disease-associated DEGs. Gene Ontology (GO) and Kyoto Encyclopedia of Genes and Genomes (KEGG) enrichment analyses were performed based on the DEGs or reversed genes and terms with FDR < 0.05 were considered significantly enriched.

### 2.10. Flow Cytometric Analysis of Skin Immune Cells

After the mice were euthanized, skin tissues from the dorsal administration area were collected and subcutaneous fat was carefully removed. The tissues were incubated with Dispase II solution (1 mg/mL; #04942078001; Roche Diagnostics GmbH, Mannheim, Germany) at 37 °C for 1 h to separate the epidermis from the dermis. The isolated dermal tissues were then minced and digested in RPMI 1640 medium containing collagenase IV (1 mg/mL; #C5138; Sigma-Aldrich, St. Louis, MO, USA) and DNase I (50 μg/mL; #DN25; Sigma-Aldrich, St. Louis, MO, USA) at 37 °C for 45 min, with gentle pipetting every 15 min to promote tissue dissociation. After digestion, the cell suspension was filtered through a 70 μm cell strainer (#352350; Corning Incorporated, Corning, NY, USA) and centrifuged at 400 g for 5 min. The cell pellet was treated with BD Pharm Lyse™ lysing buffer to remove red blood cells, washed with PBS and resuspended in BD Pharmingen™ stain buffer (FBS). For subsequent flow cytometric analysis, the cell concentration was adjusted to 4 × 10^6^ cells/mL. For the detection of helper T-cell subsets, single-cell suspensions were stimulated with BD Pharmingen™ Leukocyte Activation Cocktail with BD GolgiPlug™ at 37 °C for 4–6 h before staining. The cells were then subjected to viability staining, followed by incubation with a surface antibody cocktail containing anti-CD45, anti-CD3 and anti-CD4 at 4 °C in the dark for 30 min. After washing, the cells were fixed and permeabilized using fixation/permeabilization buffer. Intracellular staining was then performed with antibodies against IFN-γ, IL-4 and IL-17A at 4 °C in the dark for 30 min. For the detection of regulatory T cells (Tregs), single-cell suspensions were subjected to viability staining and incubated with anti-CD16/CD32 antibody as an Fc receptor blocker at 4 °C for 15 min. The cells were then stained with a surface antibody cocktail containing anti-CD45, anti-CD3, anti-CD4 and anti-CD25 at 4 °C in the dark for 30 min. After washing, the cells were fixed and permeabilized using the BD Pharmingen™ Transcription Factor Buffer Set, followed by intracellular staining with anti-Foxp3 antibody at 4 °C in the dark for 30 min. After staining, the cells were washed twice with PBS and resuspended in staining buffer for detection. All samples were analyzed using BD LSRFortessa SORP flow cytometer (BD Biosciences, San Jose, CA, USA) and the data were processed using FlowJo software (version 10).

### 2.11. Regulation of Th17/Treg Differentiation Balance in Naive CD4^+^ T Cells

To further investigate the regulatory effect of DsEXO@MTX on CD4^+^ T cell differentiation, spleen tissues were aseptically isolated from healthy C57BL/6J mice without psoriasis-like model induction. The spleens were gently ground and filtered through a 70 μm cell strainer to prepare single-cell suspensions. The cells were washed with PBS and resuspended in culture medium. Naive CD4^+^ T cells were then isolated using a magnetic bead-based sorting kit (CD4^+^CD62L^+^ T Cell Isolation Kit, Miltenyi Biotec, Bergisch Gladbach, Germany). Purified naive CD4^+^ T cells were stimulated with anti-CD3 antibody (2 μg/mL) and anti-CD28 antibody (2 μg/mL). After that, the cells were divided into eight groups. For Th17 differentiation, cells were cultured in differentiation medium containing TGF-β (2.5 ng/mL), IL-6 (15 ng/mL), anti-IFN-γ antibody (10 μg/mL) and anti-IL-4 antibody (10 μg/mL). The following four groups were included: Th17 group, Th17 + DsEXO group, Th17 + MTX group and Th17 + DsEXO@MTX group. For Treg differentiation, cells were cultured in differentiation medium containing TGF-β (1.5 ng/mL), anti-IFN-γ antibody (10 μg/mL) and anti-IL-4 antibody (10 μg/mL). The following four groups were included: Treg group, Treg + DsEXO group, Treg + MTX group and Treg + DsEXO@MTX group. In the MTX and DsEXO@MTX groups, the final concentration of MTX was maintained at 50 nM. DsEXO alone was added at an equivalent carrier concentration corresponding to that in the DsEXO@MTX group. The Th17 and Treg polarization conditions were adapted from established protocols with minor modifications [35,36]. All cells were cultured in a humidified incubator at 37 °C with 5% CO_2_ for 5 d. At the end of culture, cells from Th17 differentiation groups were stimulated with BD Pharmingen™ Leukocyte Activation Cocktail with BD GolgiPlug™ at 1 μL/mL for 4–6 h before intracellular cytokine staining. Cells from Treg differentiation groups were collected for Foxp3 staining. Flow cytometric analysis was then performed to determine the proportions of Th17 cells (CD4^+^IL-17A^+^) and Treg cells (CD4^+^CD25^+^Foxp3^+^).

### 2.12. Effect of DsEXO@MTX on STAT3 Activation in Naive CD4^+^ T Cells

Naive CD4^+^ T cells were cultured under Th17/Treg differentiation conditions with DsEXO@MTX for 5 d. For Th17 detection, cells were stimulated with BD Pharmingen™ Leukocyte Activation Cocktail with BD GolgiPlug™ at 1 μL/mL for 4–6 h, surface-stained with anti-CD4-BV605, fixed, permeabilized and intracellularly stained with anti-IL-17A-PE. For Treg detection, cells were directly surface-stained with anti-CD4-BV605 and anti-CD25-APC, then intracellularly stained with anti-Foxp3-PE. For p-STAT3 analysis, parallel unstimulated samples were surface-stained with anti-CD4-BV605, fixed, permeabilized and stained with anti-p-STAT3 (Tyr705)-FITC. Th17 (CD4^+^IL-17A^+^), Treg (CD4^+^CD25^+^Foxp3^+^) and p-STAT3 levels were quantified by flow cytometry.

### 2.13. Statistical Analysis

All data are presented as the mean ± standard deviation (SD). Comparisons between two groups were performed using an unpaired Student’s *t*-test. Comparisons among multiple groups were performed using one-way analysis of variance (ANOVA), followed by Tukey’s multiple comparisons test for post hoc analysis. A value of *p* < 0.05 was considered statistically significant. All in vitro experiments were performed in at least three independent replicates. For animal experiments, *n* = 5 mice per group, with each *n* representing an individual mouse.

## 3. Results

### 3.1. Morphological Characterization and Particle Size Determination

#### 3.1.1. Morphological Characterization and Particle Size Analysis of DsEXO

**Figure 1:**
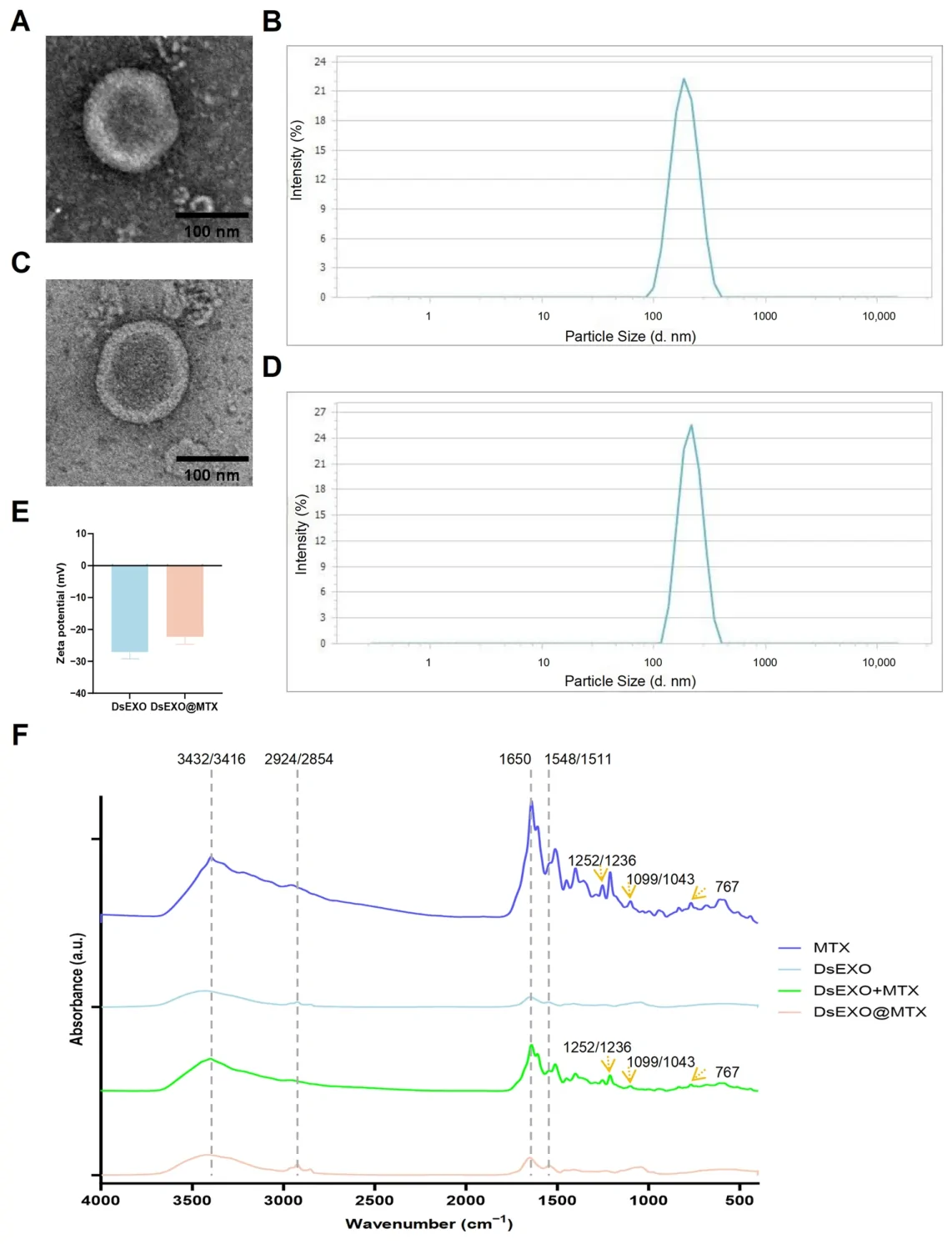
Characterization of DsEXO and DsEXO@MTX. (**A**) TEM image of DsEXO. (**B**) DLS particle size distribution of DsEXO. (**C**) TEM image of DsEXO@MTX. (**D**) DLS particle size distribution of DsEXO@MTX. (**E**) Zeta potential analysis of DsEXO and DsEXO@MTX. (**F**) FTIR spectra of MTX, DsEXO, DsEXO + MTX physical mixture and DsEXO@MTX. The main absorption bands at approximately 3432/3416, 2924/2854, 1650, 1548/1511, 1252/1236, 1099/1043 and 767 cm^−1^ were highlighted to compare the spectral features of free MTX, DsEXO, DsEXO + MTX and DsEXO@MTX. Compared with DsEXO + MTX, DsEXO@MTX showed weakened MTX-associated fingerprint peaks and slight shifts in the O–H/N–H, amide I, amide II and C–O/C–O–C regions, supporting successful MTX loading and possible non-covalent interactions between MTX and DsEXO components.

As shown in Figure 1A, DsEXO exhibited a typical disc-like morphology with relatively clear membrane boundaries, which was consistent with the morphological characteristics of exosome-like nanovesicles. The particle size distribution of DsEXO was analyzed by DLS and the average hydrodynamic diameter of DsEXO in aqueous solution was 214.70 ± 5.87 nm, with a PDI of 0.347 ± 0.006 (Figure 1B). This value was larger than the particle size observed by TEM, which may be attributed to the hydrated state of vesicles in aqueous solution and the different detection principles of TEM and DLS. Additionally, zeta potential analysis demonstrated that DsEXO exhibited a surface charge of −27.04 ± 2.17 mV, indicating a negatively charged surface. These results confirmed that the DsEXO with typical exosome-like nanovesicle morphology and nanoscale particle size was successfully isolated from the culture supernatant of Ds.

#### 3.1.2. Morphological Characterization and Particle Size Analysis of DsEXO@MTX

As shown in Figure 1C,D, DsEXO@MTX still maintained an intact vesicle-like morphology (approximately 150 nm) with relatively clear membrane boundaries after MTX loading by electroporation, indicating that the electroporation process did not cause obvious structural disruption of vesicles. DLS analysis showed that the average hydrodynamic diameter of DsEXO@MTX was 278.40 ± 3.32 nm. This value was larger than that of unloaded DsEXO and was also larger than the apparent particle size estimated from TEM images. This difference may be partly attributed to the hydrated state of vesicles and slight membrane rearrangement after MTX loading. Zeta potential analysis showed that DsEXO@MTX had a surface charge of −22.32 ± 2.27 mV (Figure 1E), indicating that the vesicles retained negative surface charge after MTX loading. Compared with unloaded DsEXO, DsEXO@MTX exhibited an increased hydrodynamic diameter, whereas TEM images confirmed that the vesicle-like structure remained intact. These results suggested that MTX was successfully loaded into DsEXO without obvious morphological disruption, supporting the successful construction of the DsEXO@MTX drug delivery system.

#### 3.1.3. FTIR Analysis

Native DsEXO showed characteristic absorption bands at 3432.46, 2923.97, 1647.74, 1548.31, 1411.40, 1242.37 and 1045.39 cm^−1^, which were mainly associated with O–H/N–H stretching, lipid C–H stretching, amide I, amide II and C–O/C–O–C vibrations, indicating the presence of protein, lipid and polysaccharide/glycoprotein components in DsEXO [37] (Figure 1F). Free MTX exhibited distinct absorption peaks at 3396.00, 1640.73, 1510.78, 1449.20, 1399.54, 1252.13, 1209.38, 1098.67, 766.91 and 608.12 cm^−1^. In the physical mixture of DsEXO and MTX (DsEXO + MTX), several MTX-related characteristic peaks remained clearly visible, particularly those at 1640.87, 1608.14, 1510.90, 1448.44, 1399.87, 1252.11, 1210.02, 1099.06, 767.26 and 608.94 cm^−1^, suggesting the retention of free or crystalline MTX features in the physical mixture. In contrast, the DsEXO@MTX spectrum largely retained the main DsEXO-associated bands, while slight shifts were observed in the O–H/N–H band from 3432.46 to 3415.90 cm^−1^, the amide I band from 1647.74 to 1651.64 cm^−1^ and the amide II band from 1548.31 to 1544.14 cm^−1^. In addition, the bands around 1242.37 and 1045.39 cm^−1^ shifted to 1235.74 and 1042.93 cm^−1^, respectively. These spectral changes indicate that MTX was successfully associated with DsEXO after loading, likely through non-covalent interactions such as hydrogen bonding, electrostatic interactions and hydrophobic interactions with lipid/protein-related functional groups. Together with the particle size, zeta potential and morphological characterization results, these findings support the successful construction of DsEXO@MTX.

#### 3.1.4. Encapsulation Efficiency and Drug Loading Content of DsEXO@MTX

The amount of unencapsulated MTX in the pooled supernatant was quantified by HPLC (Figure S1). The MTX concentration was determined to be 32.326 μg/mL, corresponding to a free MTX amount of 0.744 mg. Based on the initially added MTX amount, approximately 0.256 mg of MTX was estimated to be loaded into DsEXO. The resulting EE and estimated DLC were 25.65% and 20.41%, respectively (Table S1). These results demonstrated the successful loading of MTX into DsEXO and provided the quantitative basis for the formulation used in subsequent in vivo experiments.

#### 3.1.5. Physicochemical Characteristics of DsEXO After Storage at −80 °C

Following storage at −80 °C for 3 months and a single thaw, DsEXO exhibited a hydrodynamic diameter of 215.03 ± 10.72 nm, a PDI of 0.366 ± 0.044, and a zeta potential of −21.56 ± 0.90 mV. Freshly prepared DsEXO showed corresponding values of 214.70 ± 5.87 nm, 0.347 ± 0.006, and −27.04 ± 2.17 mV, respectively. The mean hydrodynamic diameter was generally maintained after storage and thawing, whereas a modest increase in PDI and a reduction in the magnitude of the negative zeta potential were observed. These results indicate that the overall physicochemical characteristics of DsEXO were broadly preserved, although some increase in particle-size heterogeneity and alteration in surface charge occurred (Table S2).

### 3.2. DsEXO Enhances Cellular Uptake and SKIN Delivery of FITC-MTX

#### 3.2.1. Cellular Uptake of DsEXO

**Figure 2:**
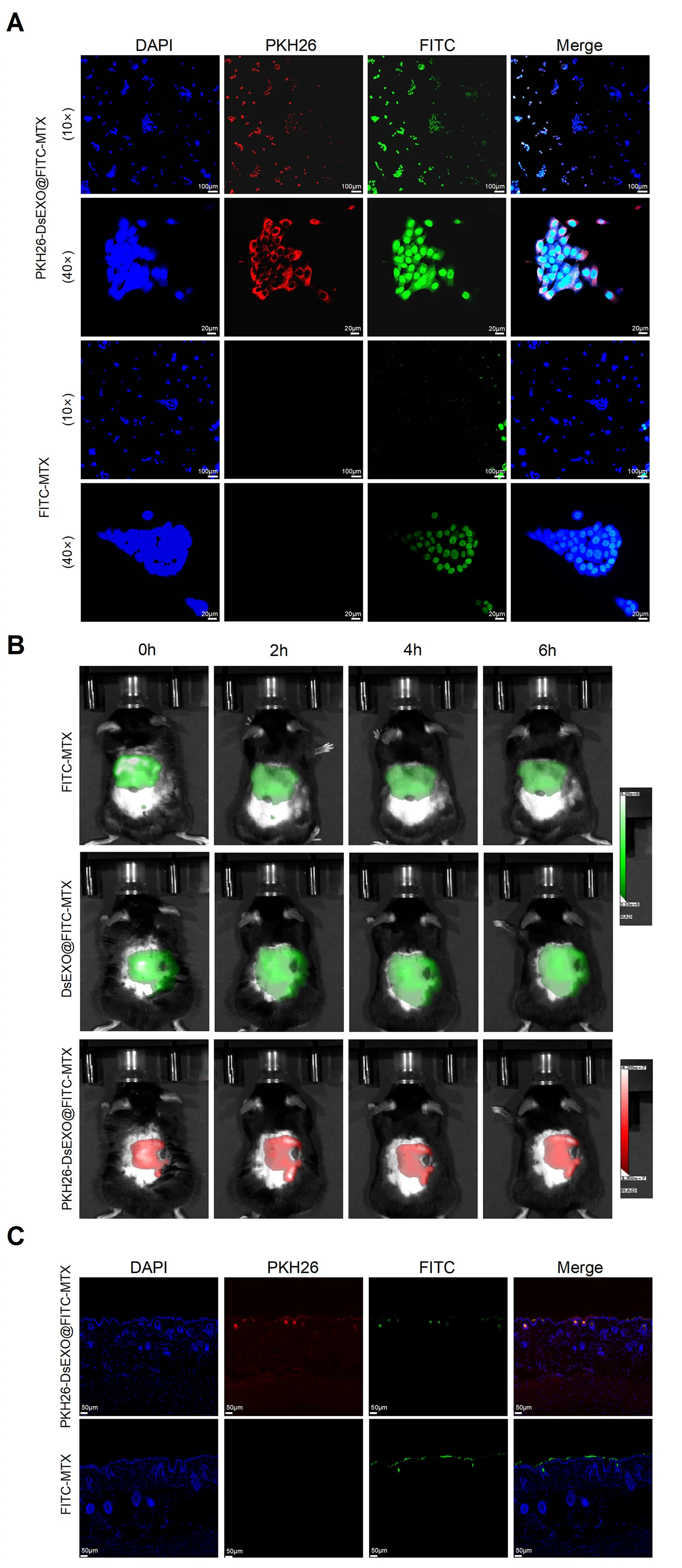
Evaluation of DsEXO-mediated cellular uptake and skin delivery of FITC-MTX. (**A**) Cellular uptake of free FITC-MTX and PKH26-DsEXO@FITC-MTX in HaCaT cells observed by confocal laser scanning microscopy. Blue, red and green fluorescence indicate nuclei stained with DAPI, PKH26-DsEXO and FITC-MTX, respectively. Scale bars: 100 µm for 10× images and 20 µm for 40× images. (**B**) In vivo fluorescence imaging of mice after topical administration of free FITC-MTX or PKH26-DsEXO@FITC-MTX at different time points. Green fluorescence represents FITC-MTX and red fluorescence represents PKH26-DsEXO. (**C**) Fluorescence distribution in frozen skin sections collected from the administration site after topical treatment, showing the skin retention and tissue penetration of free FITC-MTX and PKH26-DsEXO@FITC-MTX (scale bar = 50 µm).

Confocal laser scanning microscopy (CLSM) was used to assess the cellular uptake of FITC-MTX and DsEXO-mediated delivery of FITC-MTX in HaCaT cells. As shown in Figure 2A, FITC-MTX, PKH26-DsEXO and DAPI-stained nuclei are shown in green, red and blue, respectively. The results showed that intracellular green fluorescence was observed in the free FITC-MTX group, indicating that FITC-MTX could be taken up by HaCaT cells. Compared with free FITC-MTX, the PKH26-DsEXO@FITC-MTX group exhibited stronger intracellular green fluorescence, accompanied by red DsEXO fluorescence signals distributed within cells, suggesting efficient cellular uptake of DsEXO by HaCaT cells. The merged images further showed partial co-localization between the green FITC-MTX signal and the red PKH26-DsEXO signal in cytoplasmic and perinuclear regions, indicating DsEXO-mediated intracellular delivery of FITC-MTX. These results suggested that DsEXO possesses favorable cellular uptake capability and can enhance the intracellular delivery of small-molecule cargoes into keratinocytes, providing delivery-related evidence for the subsequent local therapeutic effects of DsEXO@MTX.

#### 3.2.2. Skin Delivery of DsEXO

In vivo imaging results showed that fluorescence signals could be observed at the skin administration site after treatment with either free FITC-MTX or PKH26-DsEXO@FITC-MTX (Figure 2B). Over 6 h observation period, the fluorescence signal in free FITC-MTX group gradually decreased and showed partial diffusion. In contrast, the PKH26-DsEXO@FITC-MTX group maintained stronger fluorescence signals at the administration site, suggesting that DsEXO loading enhanced the local retention of model cargo in skin. Meanwhile, red fluorescence signals were continuously detected at the administration site in PKH26-DsEXO@FITC-MTX group, further indicating that DsEXO carrier itself remained enriched at the administration site during the observation period. These results suggest that DsEXO contribute to improving the local retention of small-molecule cargoes at skin administration site. Since in vivo imaging mainly reflects the overall fluorescence distribution on body surface and in superficial tissues, it cannot directly determine the precise spatial distribution of cargo within skin tissues. Therefore, frozen sections of treated skin were further prepared and fluorescence microscopy was used to observe the distribution of fluorescence signals within skin tissue (Figure 2C). The fluorescence signal in free FITC-MTX group was mainly distributed on the skin surface and in the superficial epidermis, whereas PKH26-DsEXO@FITC-MTX group exhibited stronger fluorescence signals in epidermal region, with more extensive fluorescence distribution also observed in dermal region. These findings indicate that DsEXO loading facilitates the delivery of model cargo into skin tissues and improves its tissue distribution in both epidermal and dermal regions. Taken together, in vivo imaging and frozen skin section results demonstrate that the PKH26-DsEXO@FITC-MTX system exhibits superior local skin retention and enhanced tissue distribution compared with free FITC-MTX, providing experimental evidence for the subsequent topical delivery application of DsEXO@MTX.

### 3.3. DsEXO@MTX-T Ameliorates IMQ-Induced Psoriasis-Like Lesions in Mice

**Figure 3:**
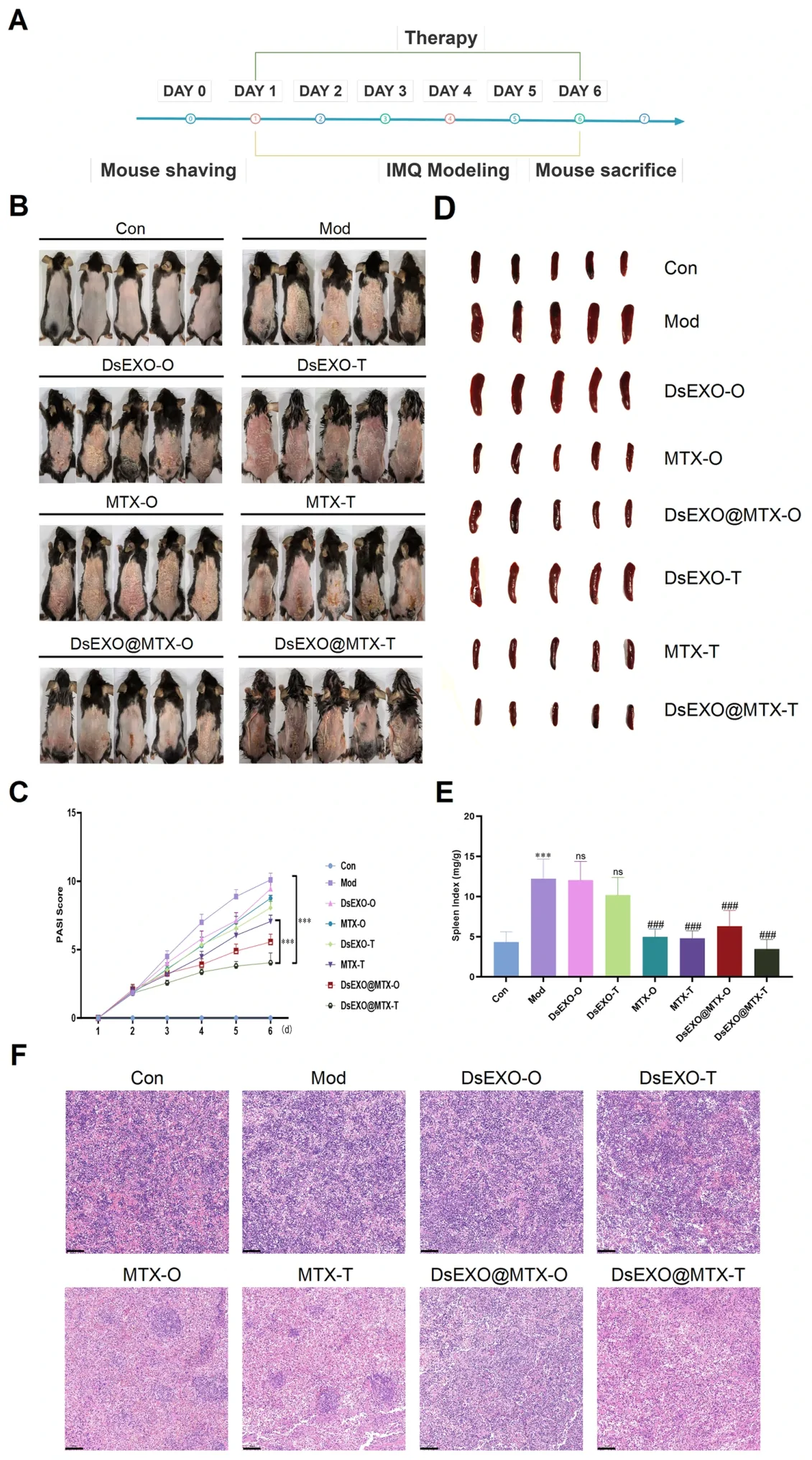
DsEXO@MTX-T alleviated IMQ-induced psoriasis-like skin lesions in mice. (**A**) Schematic illustration of animal experimental design. (**B**) Representative images of dorsal skin lesions in mice from each group. (**C**) PASI scores of dorsal skin lesions. (**D**) Representative images of spleens from each group. (**E**) Spleen index of mice in each group. (**F**) Representative H&E-stained spleen sections showing pathological changes in each group. Scale bar = 100 μm. Data are presented as the mean ± SD (*n* = 5 mice per group). ns, not significant; *** *p* < 0.001 vs. Con group; ### *p* < 0.001 vs. Mod group.

To evaluate the therapeutic efficacy of nanomedicine against psoriasis, a mouse model of IMQ-induced psoriasis-like dermatitis was established via topical application of IMQ to the dorsal skin, followed by administration of DsEXO@MTX (Figure 3A). By comparing the therapeutic efficacy of topical (T) versus oral administration (O) of DsEXO@MTX, the results showed that mice in the Mod group developed typical psoriasis-like lesions on the dorsal skin, characterized by obvious erythema, scaling and skin thickening (Figure 3B). Compared with Mod group, treatment with DsEXO alone showed limited improvement in skin lesions, whereas MTX-O, MTX-T, DsEXO@MTX-O and DsEXO@MTX-T treatments alleviated erythema and scaling to varying degrees. Among all treatment groups, DsEXO@MTX-T exhibited the most pronounced therapeutic effect. Disease severity was quantitatively evaluated using a modified PASI scoring system (Figure 3C). Compared with MTX-O group, modified PASI score was significantly decreased in the DsEXO@MTX-O group. In addition, DsEXO@MTX-T further reduced the modified PASI score compared with MTX-T and DsEXO@MTX-O group, indicating that DsEXO-mediated delivery enhanced the overall therapeutic efficacy of topical MTX. Furthermore, IMQ induction resulted in marked spleen enlargement in mice (Figure 3D), whereas DsEXO@MTX-T treatment effectively alleviated splenomegaly and significantly reduced the spleen index (Figure 3E). Histological observation showed that the spleen in Con group exhibited relatively intact architecture, with well-defined white pulp and a clear boundary between red pulp and white pulp (Figure 3F). By contrast, splenic tissue from Mod group exhibited disorganized architecture with blurred demarcation between red and white pulp and a marked reduction in white pulp area, indicative of IMQ-induced systemic immune dysregulation. Following DsEXO@MTX-T treatment, splenic architecture was partially restored, as evidenced by more distinct white pulp regions and improved tissue organization. Collectively, these findings demonstrate that DsEXO@MTX-T, especially via topical administration, effectively attenuates both IMQ-induced psoriasis-like skin lesions and systemic immune perturbations.

### 3.4. DsEXO@MTX-T Inhibits IMQ-Induced Keratinocyte Proliferation in Mice

**Figure 4:**
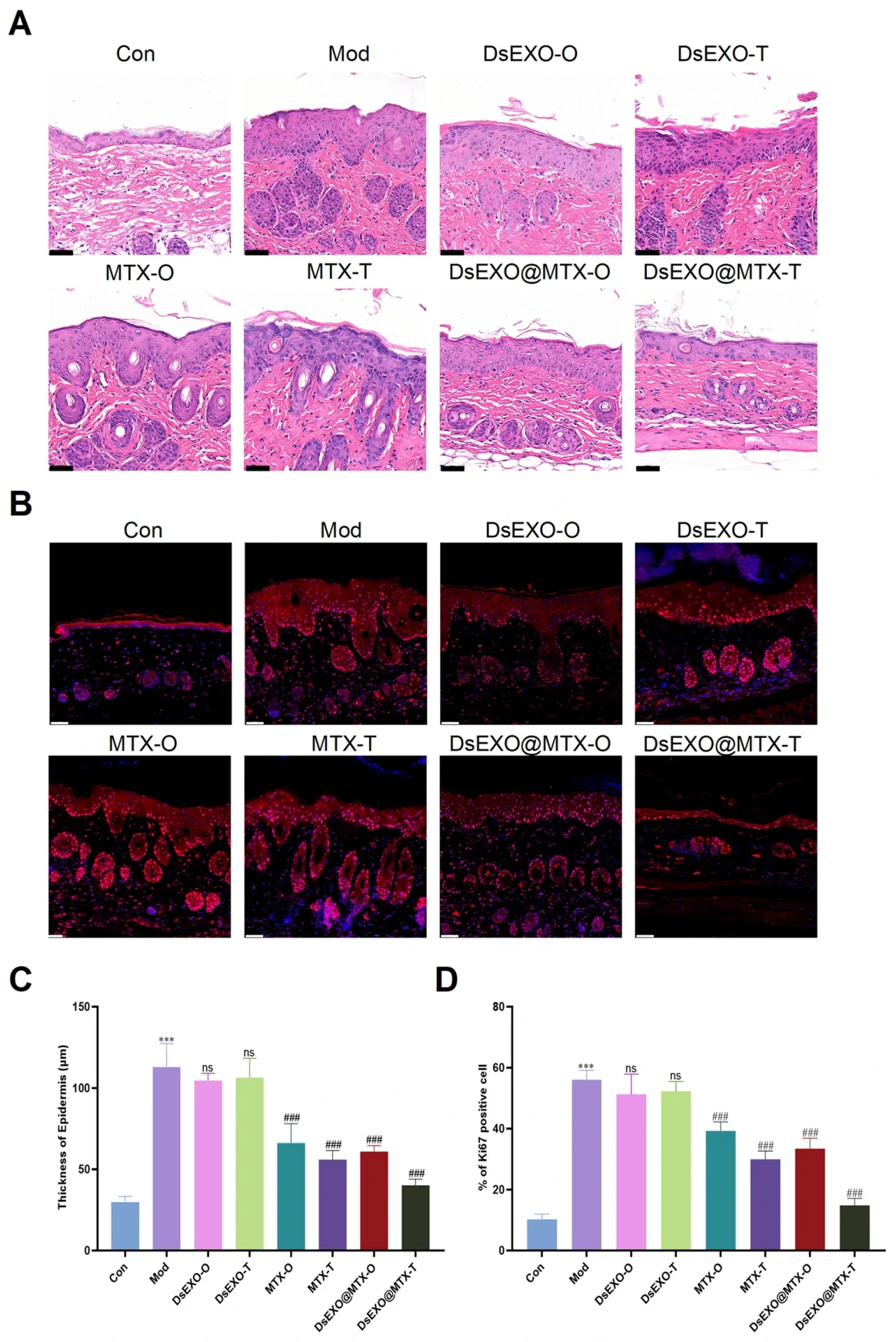
DsEXO@MTX-T alleviates epidermal hyperplasia and suppresses keratinocyte hyperproliferation in IMQ-induced psoriasis-like skin lesions. (**A**) Representative H&E-stained skin sections from each group. (**B**) Representative Ki-67 immunofluorescence staining of skin lesions in each group. Ki-67 is shown in red and nuclei are counterstained with DAPI in blue. (**C**) Quantification of epidermal thickness. (**D**) Quantification of Ki-67-positive cells. Scale bar = 50 μm. Data are presented as the Mean ± SD. ns, not significant; *** *p* < 0.001 vs. Con group; ### *p* < 0.001 vs. Mod group.

As shown in Figure 4A, the epidermis of mice in Con group was thin and smooth, with an intact structure and no obvious inflammatory cell infiltration. In contrast, mice in Mod group exhibited typical psoriasis-like pathological changes, including marked epidermal thickening, acanthosis, parakeratosis and inflammatory cell infiltration in the dermis, indicating the successful establishment of psoriasis-like mouse model. Compared with Mod group, skin pathological changes were improved to varying degrees in all treatment groups except the DsEXO-alone groups. Among them, the DsEXO@MTX-T group showed the most pronounced improvement, with markedly reduced epidermal thickness (*p* < 0.001) (Figure 4C), accompanied by only mild keratinization and limited inflammatory cell infiltration, suggesting that DsEXO@MTX-T effectively ameliorated the pathological skin changes in psoriasis-like mice.

Ki-67 immunofluorescence staining was further performed to evaluate keratinocyte proliferation (Figure 4B). In Con group, only scattered Ki-67-positive cells were observed in the basal layer of epidermis. In Mod group, the number of Ki-67-positive cells was markedly increased and the positive staining extended from basal layer to spinous and granular layers, indicating that epidermal cells in psoriasis-like mice were in a highly proliferative state. Compared with Mod group, the number of Ki-67-positive cells was reduced to varying degrees in all treatment groups except the DsEXO-alone groups and positive signals were mainly restricted to the basal layer. Notably, DsEXO@MTX-T group exhibited the most pronounced inhibitory effect, with the number of Ki-67-positive cells decreasing to a level similar to that in Con group and only a few scattered positive cells observed in basal layer. DsEXO@MTX-T treatment significantly reduced the proportion of Ki-67-positive cells (*p* < 0.001, Figure 4D), suggesting that this treatment effectively reduced abnormal keratinocyte proliferation in psoriasis-like mice.

### 3.5. DsEXO@MTX-Mediated STAT3-Th17/Treg Axis Analysis

**Figure 5:**
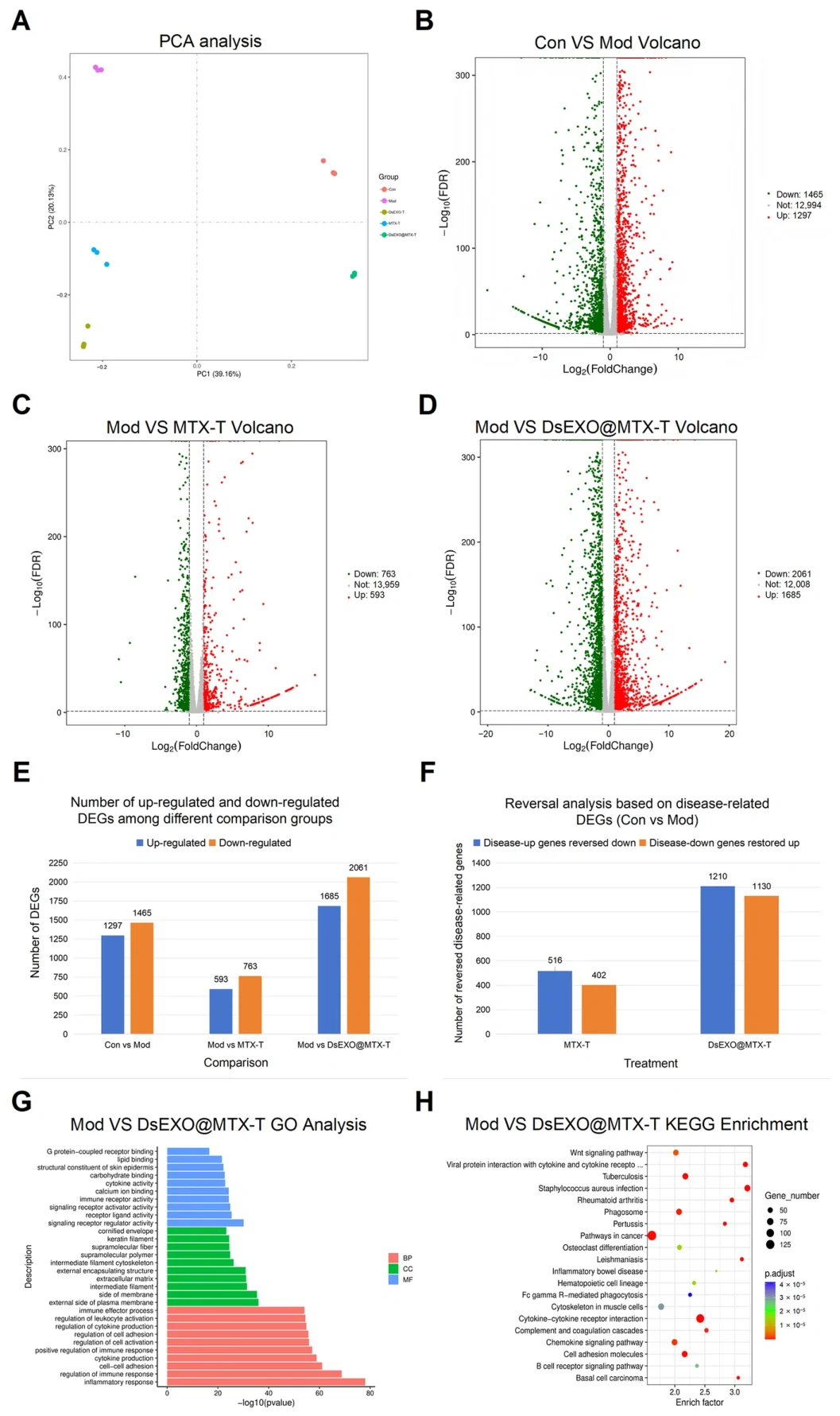
Transcriptomic analysis of potential therapeutic mechanisms. (**A**) PCA showing global transcriptomic separation among the five groups. (**B**) Volcano plot of DEGs between Con and Mod groups. (**C**) Volcano plot of DEGs between Mod and MTX-T groups. (**D**) Volcano plot of DEGs between Mod and DsEXO@MTX-T groups. (**E**) Numbers of up-regulated and down-regulated DEGs across the three comparisons. (**F**) Reversal analysis based on disease-related DEGs (Con vs. Mod). (**G**) GO enrichment analysis of DEGs between Mod and DsEXO@MTX-T groups. (**H**) KEGG pathway enrichment analysis of DEGs between Mod and DsEXO@MTX-T groups. Dashed lines in the volcano plots indicate the thresholds of |log_2_ fold change| ≥ 1 and FDR < 0.05.

To elucidate the molecular mechanisms underlying the therapeutic effects of DsEXO@MTX-T in IMQ-induced psoriasis-like inflammation, RNA-seq analysis was performed on skin tissue samples from the Con, Mod, DsEXO-T, MTX-T and DsEXO@MTX-T groups. PCA revealed clear global transcriptomic separation among groups, with PC1 and PC2 explaining 39.16% and 20.13% of total variance, respectively (Figure 5A). Con and Mod groups were distinctly separated along PC1, confirming substantial IMQ-induced transcriptomic alterations. Notably, DsEXO@MTX-T samples shifted toward the Con group along PC1 and were clearly distinguished from the Mod, DsEXO-T and MTX-T groups, whereas MTX-T and DsEXO-T samples remained largely in negative PC1 region, indicating that DsEXO@MTX-T exerted a more pronounced transcriptomic corrective effect. However, residual separation along PC2 suggested incomplete restoration to the normal state.

Differential expression analysis identified 2762 DEGs between Con and Mod groups (1297 upregulated and 1465 downregulated; Figure 5B), reflecting extensive gene expression remodeling involving inflammatory responses, chemokine activity, immune cell infiltration and abnormal keratinocyte activation, consistent with typical psoriasis-like features. Compared with Mod group, MTX-T induced 1356 DEGs (Figure 5C), indicating transcriptional regulation after MTX-T treatment, whereas DsEXO@MTX-T induced 3746 DEGs (Figure 5D), suggesting a broader regulatory effect. The numbers of upregulated and downregulated DEGs across the three comparisons are summarized in Figure 5E. Disease reversal analysis further confirmed the superiority of DsEXO@MTX-T (Figure 5F). MTX-T reversed 918 disease-associated genes (33.2% of total Con vs. Mod DEGs), while DsEXO@MTX-T reversed 2340 genes (84.7%), including 1210 downregulated model-upregulated genes and 1130 restored model-downregulated genes. Among 858 commonly reversed genes, DsEXO@MTX-T showed greater reversal magnitude for 761 (88.7%). Additionally, 1482 genes were uniquely reversed by DsEXO@MTX-T versus only 60 by MTX-T.

GO and KEGG enrichment analyses of DsEXO@MTX-T-regulated DEGs highlighted biological processes and pathways related to inflammatory response, immune regulation, cytokine production, chemokine activity, cell adhesion and epidermal remodeling (Figure 5G,H). Key enriched pathways included cytokine-cytokine receptor interaction, chemokine signaling pathway, IL-17 signaling pathway, JAK–STAT signaling pathway, complement and coagulation cascades, B cell receptor signaling pathway and Wnt signaling pathway. These enriched immune-inflammatory pathways are closely associated with psoriasis-like skin inflammation and indicate that DsEXO@MTX-T markedly modulates local inflammatory networks and epidermal pathological remodeling. Notably, Stat3 itself did not meet the predefined threshold for significant differential expression, although its transcript level showed a decreasing trend after DsEXO@MTX-T treatment compared with Mod group. Therefore, RNA-seq data suggest that the therapeutic effect of DsEXO@MTX-T may be associated with regulation of STAT3-related inflammatory signaling rather than a marked alteration in Stat3 transcription. Together with the subsequent reduction in STAT3 phosphorylation and restoration of the Th17/Treg balance, these transcriptomic findings provide a rationale for further investigation of the STAT3–Th17/Treg axis as a potential mechanism underlying the therapeutic effect of DsEXO@MTX-T.

### 3.6. DsEXO@MTX Restores Th17/Treg Imbalance in Psoriatic Skin

**Figure 6:**
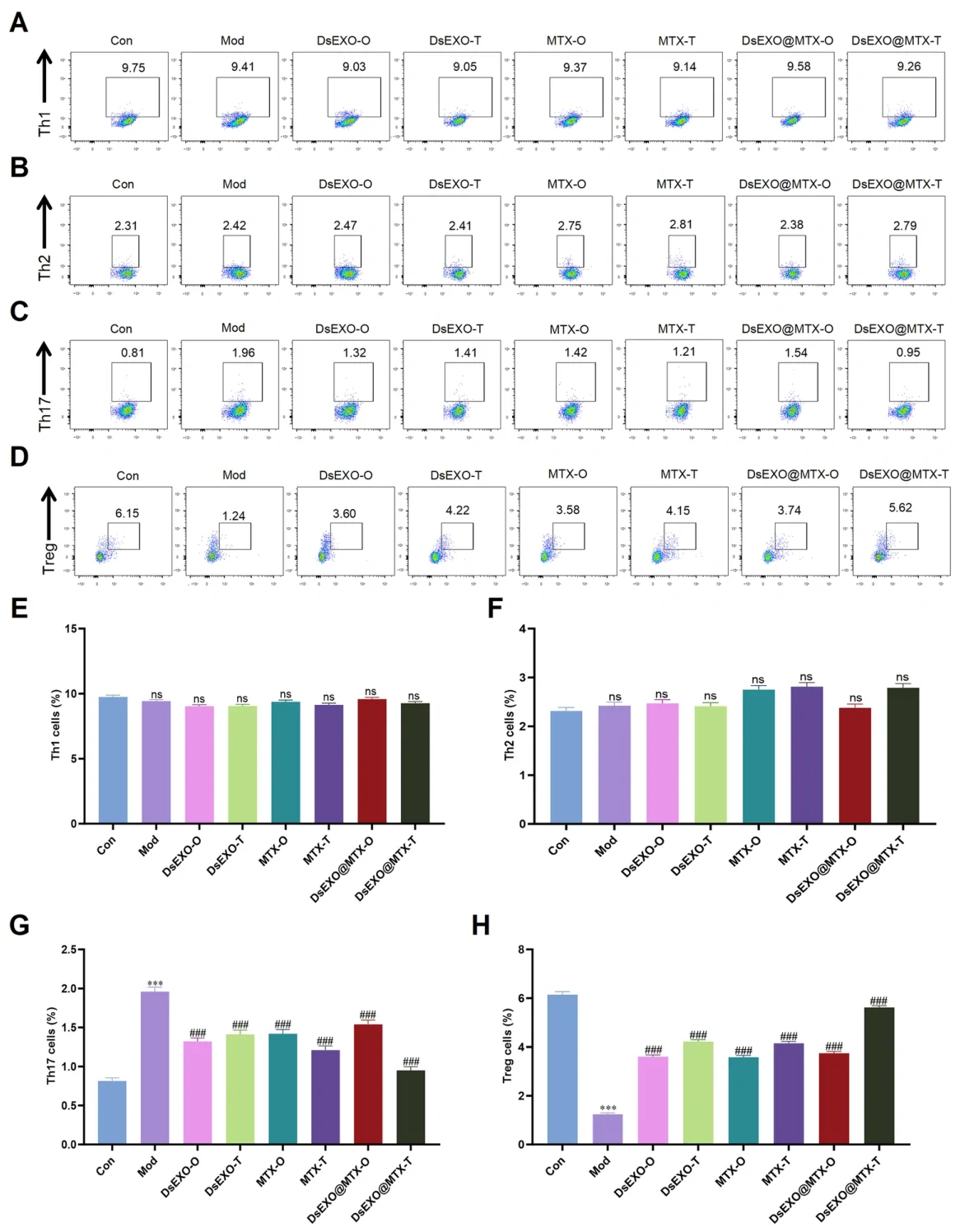
Effects of DsEXO@MTX on Th1, Th2, Th17 and Treg cell populations in IMQ-induced psoriasis-like mice. (**A**) Representative flow cytometry plots of Th1 cells in each group. (**B**) Representative flow cytometry plots of Th2 cells in each group. (**C**) Representative flow cytometry plots of Th17 cells in each group. (**D**) Representative flow cytometry plots of Treg cells in each group. (**E**–**H**) Quantitative analysis of Th1, Th2, Th17 and Treg cell percentages, respectively. Pseudocolors in the flow cytometry plots indicate event density, whereas the colors of the bars correspond to the experimental groups indicated on the x-axis. Data are presented as Mean ± SD, *n* = 3. ns, not significant; *** *p* < 0.001 vs. Con group; ### *p* < 0.001 vs. Mod group.

RNA-seq analysis identified significant enrichment of immune-inflammatory pathways, including pathways related to cytokine signaling and Th17-associated responses. Based on these findings, helper T-cell subsets in lesional skin were further examined by flow cytometry. Accordingly, we quantified the proportions of Th1, Th2, Th17 and Treg cells in murine skin lesions using flow cytometry to evaluate the effects of different interventions on the immune microenvironment in IMQ-induced psoriasis-like mice (Figure 6). Compared with the Con group, the proportions of Th1 and Th2 cells in the Mod group showed no significant changes (*p* > 0.05), whereas the proportion of Th17 cells was significantly increased and the proportion of Treg cells was significantly decreased (*p* < 0.01), suggesting that the psoriasis-like mice model was mainly characterized by local Th17/Treg immune imbalance in the skin. Compared with the Mod group, DsEXO@MTX-T exhibited the most pronounced immunomodulatory effect, as evidenced by a significant reduction in the proportion of Th17 cells and an increase in the proportion of Treg cells, with superior regulatory effects compared with the other treatment groups (*p* < 0.01). The frequencies of Th17 and Treg cells were both substantially normalized, approaching physiological levels. In contrast, the proportions of Th1 and Th2 cells were not markedly affected by the different treatments. Taken together, these results indicate that DsEXO@MTX-T primarily restored local Th17/Treg immune balance in the skin of psoriatic mice by reducing the proportion of Th17 cells and increasing the proportion of Treg cells.

### 3.7. Effects of DsEXO@MTX on STAT3 Phosphorylation and Th17/Treg Differentiation

**Figure 7:**
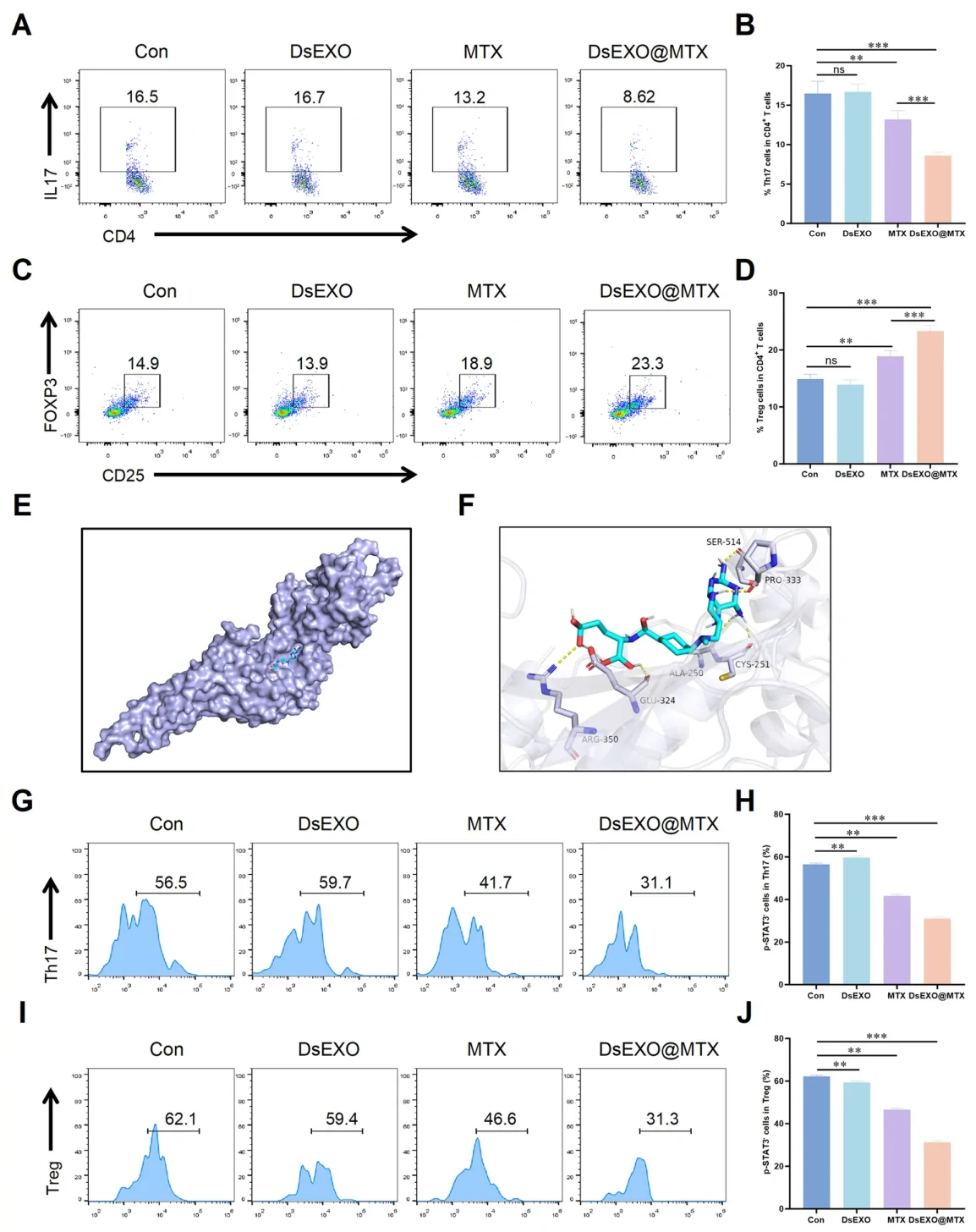
DsEXO@MTX regulates CD4^+^ T cell differentiation into Th17/Treg cells and suppresses STAT3 signaling activation in vitro. (**A**) Representative flow cytometry plots of CD4^+^IL-17A^+^ Th17 cells under Th17-differentiation conditions. (**B**) Quantitative analysis of Th17 cell percentages among CD4^+^ T cells. (**C**) Representative flow cytometry plots of CD4^+^CD25^+^Foxp3^+^ Treg cells under Treg-differentiation conditions. (**D**) Quantitative analysis of Treg cell percentages among CD4^+^ T cells. (**E**) Overall binding conformation of MTX with STAT3 protein. (**F**) Enlarged view of MTX within binding pocket of STAT3. (**G**) Representative histogram plots showing p-STAT3 expression in Th17 cells. (**H**) Quantitative analysis of the percentage of p-STAT3-positive cells in Th17 cells. (**I**) Representative histogram plots showing p-STAT3 expression in Treg cells. (**J**) Quantitative analysis of the percentage of p-STAT3-positive cells in Treg cells. The colors of the bars correspond to the experimental groups indicated on the x-axis, and the blue-filled histograms represent p-STAT3 fluorescence distributions. Data are presented as Mean ± SD, *n* = 3. ns, not significant; ** *p* < 0.01, *** *p* < 0.001.

In Th17 differentiation system, the proportions of CD4^+^IL-17A^+^ Th17 cells in Con, DsEXO, MTX and DsEXO@MTX groups were 16.5%, 16.7%, 13.2% and 8.62%, respectively. These findings demonstrate that DsEXO alone exerts no discernible effect on Th17 differentiation, whereas MTX partially inhibited Th17 differentiation (*p* < 0.01 vs. Con group). Notably, DsEXO@MTX exhibited the most significant inhibitory effect on Th17 differentiation (*p* < 0.001 vs. MTX group; *p* < 0.001 vs. Con group), suggesting that DsEXO enhanced the inhibitory effect of MTX on Th17 differentiation. In Treg differentiation system, the proportions of CD4^+^CD25^+^Foxp3^+^ Treg cells in four groups were 14.9%, 13.9%, 18.9% and 23.3%, respectively. Compared with Con group, both MTX and DsEXO@MTX increased the proportion of Treg cells (both *p* < 0.001), with the most pronounced increase observed in DsEXO@MTX group (*p* < 0.001 vs. MTX group). These results indicate that DsEXO@MTX can directly regulate the differentiation direction of naive CD4^+^ T cells by inhibiting Th17 differentiation and promoting Treg differentiation, thereby improving Th17/Treg differentiation imbalance (Figure 7A–D).

Molecular docking analysis showed that MTX could bind to the potential binding pocket of STAT3 protein (Figure 7E,F). AutoDock Vina (version 1.2.5) results showed that binding energy of the optimal docking conformation was −7.267 kcal/mol, indicating a favorable binding affinity between MTX and STAT3. Interaction analysis further revealed that MTX interacted with multiple amino acid residues surrounding the STAT3 binding pocket mainly through hydrogen bonds, including Arg350, Glu324, Ala250, Cys251, Pro333 and Ser514. In docking diagram, hydrogen-bond interactions are indicated by yellow dashed lines. These results suggest that MTX can form a relatively stable complex with STAT3, providing molecular-level theoretical evidence for the potential regulation of STAT3-related signaling pathways by MTX. During Th17 differentiation, the proportions of p-STAT3-positive cells in Con, DsEXO, MTX and DsEXO@MTX groups were 56.5%, 59.7%, 41.7% and 31.1%, respectively (Figure 7G–J). DsEXO treatment alone had no significant effect on p-STAT3 expression. In contrast, MTX group showed a significant decrease in p-STAT3 levels (*p* < 0.05 vs. Con group), while the DsEXO@MTX group exhibited the most pronounced inhibitory effect (*p* < 0.01 vs. MTX group). During Treg differentiation, the proportions of p-STAT3-positive cells in four groups were 62.1%, 59.4%, 46.6% and 33.3%, respectively. A similar trend was observed, with the DsEXO@MTX group showing the strongest inhibitory effect on STAT3 activation (*p* < 0.05 vs. MTX group). These findings confirm that DsEXO@MTX exerts its anti-psoriatic effect via STAT3-mediated modulation of Th17/Treg cell differentiation.

### 3.8. Biosafety Evaluation

**Figure 8:**
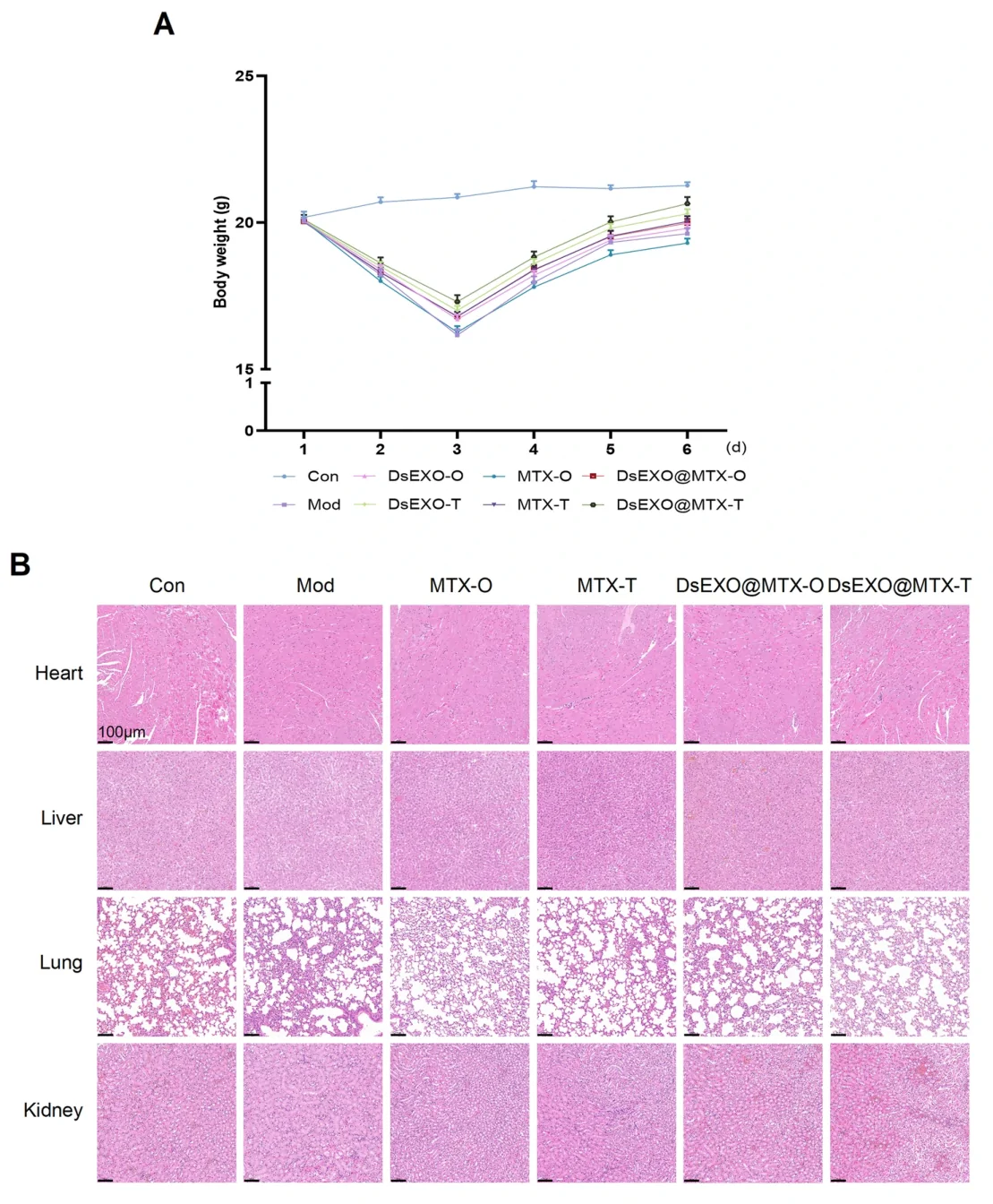
In vivo biosafety evaluation of DsEXO@MTX. (**A**) Body weight changes in mice during the 6-day treatment period. (**B**) Representative H&E-stained images of major organs, including the heart, liver, lung and kidney, from different treatment groups. Scale bar = 100 μm.

To evaluate the in vivo biosafety of DsEXO@MTX, body weight changes were monitored daily during the 6-day treatment period and major organs, including the heart, liver, lung and kidney, were collected at the end of experiment for H&E staining analysis. The results showed that body weight remained relatively stable in Con group, whereas IMQ-treated groups exhibited a transient decrease in body weight during the early stage of model induction, followed by gradual recovery. By day 6, no sustained body weight loss or visible signs of emaciation were observed in any treatment group (Figure 8A), suggesting that the treatments did not induce apparent systemic adverse effects. Further histopathological examination showed that the heart, liver, lung and kidney tissues from the treatment groups maintained intact tissue architecture and generally normal cellular arrangement, with no obvious inflammatory cell infiltration, necrosis, hemorrhage or structural disruption (Figure 8B). These results indicate that DsEXO@MTX did not cause marked body weight abnormalities or observable histological damage to major organs at the tested dose, suggesting favorable preliminary in vivo biosafety.

## 4. Discussion

MTX, a well-established systemic agent for psoriasis, is clinically limited by insufficient lesional accumulation and systemic toxicity risks, which topical administration can address by enhancing local drug exposure while minimizing systemic distribution. Nevertheless, the thickened stratum corneum, abnormal epidermal architecture and inflammatory microenvironment of psoriatic skin may restrict the retention, penetration and cellular uptake of conventional topical formulations. To overcome these issues, exosome (EXO)-based delivery represents a promising strategy for topical drug administration. However, the broad clinical application of conventional EXOs remains restricted by limited natural availability, low production yield, laborious isolation and purification procedures, batch-to-batch heterogeneity, insufficient standardization and challenges in scalable manufacturing and quality control. Therefore, DsEXO@MTX was constructed in this study as a natural nanovesicle-mediated topical delivery system for psoriasis therapy. The results demonstrated that under the tested conditions, topical DsEXO@MTX showed greater therapeutic efficacy than free MTX administered topically or orally in an IMQ-induced psoriasis-like dermatitis model. Topical DsEXO@MTX markedly alleviated erythema, scaling and skin thickening, reduced modified PASI scores, attenuated epidermal hyperplasia and inflammatory infiltration, inhibited abnormal keratinocyte proliferation and decreased the spleen index. Empty DsEXO showed only limited therapeutic effects, suggesting that the efficacy of DsEXO@MTX was mainly associated with the delivery and activity of MTX, although a contribution from the carrier itself cannot be excluded. These findings suggest that DsEXO-mediated topical delivery can enhance the local therapeutic performance of MTX against psoriasis-like skin inflammation and epidermal pathological changes. Mechanistically, the therapeutic effects of DsEXO@MTX were associated with reduced STAT3 phosphorylation and restoration of the Th17/Treg balance.

Transcriptomic analysis showed that DsEXO@MTX-T altered immune-inflammatory and epidermal remodeling programs, including cytokine–cytokine receptor interactions, chemokine signaling, IL-17 signaling and JAK–STAT signaling. Cytokine and chemokine pathways are closely associated with inflammatory-cell recruitment in psoriatic lesions [5,6,8], whereas IL-17 signaling promotes keratinocyte activation, inflammatory mediator production and epidermal hyperplasia [5,6,7]. JAK–STAT signaling links cytokines such as IL-6 and IL-23 to STAT3 activation and Th17/Treg dysregulation [9,10,11,12], while complement-related signaling may also participate in local inflammatory regulation [38]. Consistent with these pathway-level findings, flow cytometric analysis of lesional skin showed that IMQ increased the proportion of Th17 cells and decreased the proportion of Treg cells, whereas Th1 and Th2 proportions were not markedly altered. Topical DsEXO@MTX reversed the Th17/Treg imbalance. In vitro, DsEXO@MTX inhibited Th17 differentiation, promoted Treg differentiation and reduced the proportion of p-STAT3-positive CD4^+^ T cells more effectively than free MTX at an equivalent MTX concentration, supporting a contribution of vesicle-mediated delivery to the immunomodulatory activity of MTX. Collectively, these findings support an association between DsEXO@MTX treatment, reduced STAT3 phosphorylation and restoration of the Th17/Treg balance. However, the enrichment results indicate pathway-level associations rather than direct evidence of pathway activation or causal regulation. Moreover, although Stat3 did not meet the predefined threshold for differential expression, this finding is not inconsistent with the reduced p-STAT3 levels because STAT3 activity is primarily regulated through phosphorylation rather than changes in total transcript abundance. Molecular docking provided additional computational support for a possible MTX–STAT3 interaction, although direct intracellular binding and the status of STAT3 as a molecular target of MTX remain to be experimentally established.

The emergence of advanced drug delivery technologies has opened new therapeutic avenues in psoriasis management. Among these, lipid-based nanocarriers, microneedle-assisted systems and electrospun patches have been explored because of their potential to improve local drug deposition and provide controlled release [16,17,20]. Topical treatment remains an important component of psoriasis management, particularly for localized disease [15]. Another important finding of this study is that topical DsEXO@MTX showed better therapeutic performance than oral DsEXO@MTX. Oral MTX exposure is influenced by gastrointestinal absorption and pharmacokinetic variability, while systemic administration distributes the drug beyond lesional skin [39]. In contrast, topical DsEXO@MTX can be directly applied to psoriatic lesions, where DsEXO-mediated retention and cellular uptake of fluorescent cargoes may enhance local drug exposure. The reduced spleen index and improved splenic pathological changes observed after DsEXO@MTX-T treatment also suggest that local treatment may alleviate systemic immune abnormalities associated with IMQ-induced psoriasis-like inflammation. These findings support the potential of topical nanovesicle-mediated MTX delivery to improve local therapeutic efficacy, although a reduction in systemic MTX exposure was not directly demonstrated. Similar efforts to enhance topical MTX delivery have explored nanolipid carriers, transferosome-loaded microneedle patches and electrospun patches [40,41,42]. Because these formulations were not directly compared in the present study, the current findings indicate the therapeutic potential of DsEXO@MTX rather than its superiority over existing topical delivery systems.

However, some limitations still remain in this study. First, although fluorescence imaging and frozen skin sections indicated improved local retention and tissue distribution of DsEXO-loaded fluorescent cargoes, quantitative MTX pharmacokinetics, drug release, cutaneous deposition and transdermal permeation were not evaluated. These issues should be further examined using validated IVRT/IVPT methods and ex vivo human psoriatic skin to determine whether DsEXO@MTX truly enhances local drug accumulation while reducing systemic exposure. Second, the involvement of STAT3 was supported by RNA-seq, p-STAT3 measurements and T-cell differentiation assays, but direct causal evidence using pharmacological inhibition or genetic manipulation is still lacking. In addition, representative differentially expressed genes were not independently validated, and the bulk RNA-seq data cannot distinguish changes in cell composition from cell-specific transcriptional regulation. The protein and miRNA cargoes and immunogenicity of DsEXO also require further investigation. Although standardized preparation procedures were used, batch-to-batch reproducibility, long-term stability and scale-up feasibility were not systematically assessed; EV production yield, purification efficiency and quality-control consistency remain important translational considerations. Finally, the acute IMQ-induced model reproduces several inflammatory and epidermal features of psoriasis but does not fully capture the chronic, recurrent and heterogeneous nature of human disease [43,44]. Future studies should therefore employ chronic or recurrent models and further evaluate human skin penetration, formulation stability, safety, scalability and comparative efficacy.

## 5. Conclusions

In conclusion, this study developed DsEXO@MTX as a natural DsEXO-based topical delivery system for MTX treatment of psoriasis-like dermatitis. DsEXO@MTX retained an intact vesicular morphology, with a mean hydrodynamic diameter of 278.40 ± 3.32 nm and a zeta potential of −22.32 ± 2.27 mV. DsEXO-mediated delivery enhanced the cellular uptake and prolonged the local retention and epidermal–dermal distribution of FITC-MTX-associated fluorescence. In the IMQ-induced mouse model, topical DsEXO@MTX produced the most pronounced improvement among the tested treatments, reducing lesion severity, epidermal hyperplasia, Ki-67-positive keratinocytes and splenomegaly. In vitro, DsEXO@MTX reduced Th17 differentiation from 16.5% to 8.62% and increased Treg differentiation from 14.9% to 23.3%. The proportions of p-STAT3-positive cells were also reduced from 56.5% to 31.1% under Th17-polarizing conditions and from 62.1% to 33.3% under Treg-polarizing conditions. Collectively, these findings indicate that topical DsEXO@MTX improves local MTX-associated cargo delivery and alleviates psoriasis-like inflammation, with its immunomodulatory effects being associated with reduced STAT3 phosphorylation and restoration of the Th17/Treg balance.

## Data Availability

All data in this article are included within the article. It can also be requested from the corresponding authors or first authors.

## Supplementary Materials

The following supporting information can be downloaded at: https://www.mdpi.com/article/10.3390/pharmaceutics18091085/s1, Figure S1: Representative HPLC chromatograms of MTX. (**A**) MTX standard solution at 50 ug/mL, with a retention time of 30.620 min. (**B**) Unencapsulated MTX in the pooled supernatant obtained after purification of DsEXO@MTX, with a retention time of 30.962 min; Table S1: Calculation of encapsulation efficiency and estimated drug loading content of DsEXO@MTX; Table S2: Physicochemical characteristics of freshly prepared and post-thaw DsEXO after storage at −80°C for 3 months.
