# Supplementary material for: Topical Dunaliella salina-Derived Exosome Loaded with Methotrexate Alleviates Psoriasis-like Inflammation via STAT3-Dependent Th17/Treg Balance
Source: Pharmaceutics. 2026 Aug 28;18(9):1085. doi: 10.3390/pharmaceutics18091085 (PMC13610521; doi:10.3390/pharmaceutics18091085)
Supplement: Supplementary file 1 [file pharmaceutics-18-01085-s001.zip › Figure S1.pdf]

S1A

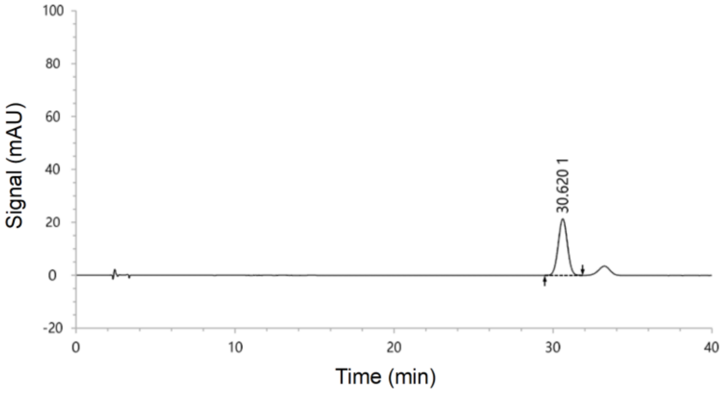

S1B

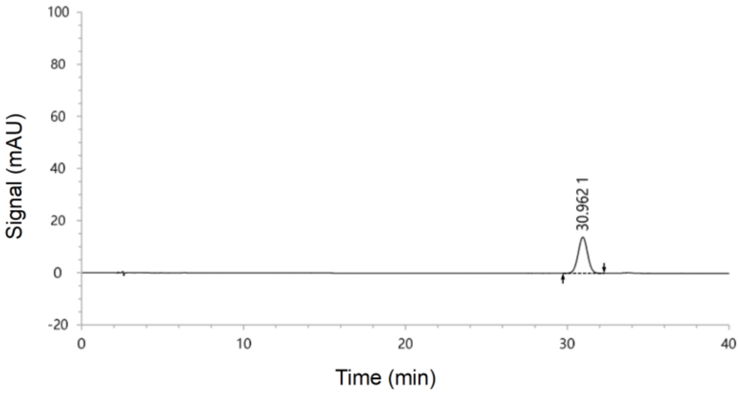

Figure S1. Representative HPLC chromatograms of MTX. **(A)** MTX standard solution at 50 ug/mL, with a retention time of 30.620 min. **(B)** Unencapsulated MTX in the pooled supernatant obtained after purification of DsEXO@MTX, with a retention time of 30.962 min.
