# Supplementary material for: Topical Dunaliella salina-Derived Exosome Loaded with Methotrexate Alleviates Psoriasis-like Inflammation via STAT3-Dependent Th17/Treg Balance
Source: Pharmaceutics. 2026 Aug 28;18(9):1085. doi: 10.3390/pharmaceutics18091085 (PMC13610521; doi:10.3390/pharmaceutics18091085)
Supplement: Supplementary file 1 [file pharmaceutics-18-01085-s001.zip › Table S1-S2.pdf]

Table S1. Calculation of encapsulation efficiency and estimated drug loading content of DsEXO@MTX

Parameter	Symbol or calculation	Value
Initial amount of MTX	$M_{\mathrm{MTX,initial}}$	1 mg
Initial amount of DsEXO	$M_{\mathrm{DsEXO,initial}}$	1 mg
MTX:DsEXO feeding ratio	—	1:1 (w/w)
MTX concentration in pooled supernatant	$C_{\mathrm{MTX,free}}$	32.326 μg/mL
Final volume of pooled supernatant	$V_{\mathrm{supernatant}}$	23 mL
Amount of unencapsulated MTX	$M_{\mathrm{MTX,free}} = C_{\mathrm{MTX,free}} \times V_{\mathrm{supernatant}}$	0.744 mg
Estimated amount of MTX loaded into DsEXO	$M_{\mathrm{MTX,loaded}} = M_{\mathrm{MTX,initial}} - M_{\mathrm{MTX,free}}$	0.256 mg
Encapsulation efficiency	$\mathrm{EE(\%)} = (M_{\mathrm{MTX,initial}} - M_{\mathrm{MTX,free}}) / M_{\mathrm{MTX,initial}} \times 100\%$	25.65%
Estimated drug loading content	$\mathrm{DLC(\%)} = M_{\mathrm{MTX,loaded}} / (M_{\mathrm{MTX,loaded}} + M_{\mathrm{DsEXO,initial}}) \times 100\%$	20.41%
Estimated MTX:DsEXO ratio in final formulation	$M_{\mathrm{MTX,loaded}} : M_{\mathrm{DsEXO,initial}}$	1:3.90

Table S2. Physicochemical characteristics of freshly prepared and post-thaw DsEXO after storage at −80°C for 3 months

Sample condition	Hydrodynamic diameter (nm)	PDI	Zeta potential (mV)
Freshly prepared DsEXO	214.70 ± 5.87	0.347 ± 0.006	−27.04 ± 2.17
DsEXO stored at −80 °C for 3 months and thawed once	215.03 ± 10.72	0.366 ± 0.044	−21.56 ± 0.90
